# Comprehensive Analysis of Codon Usage on Rabies Virus and Other Lyssaviruses

**DOI:** 10.3390/ijms19082397

**Published:** 2018-08-14

**Authors:** Xu Zhang, Yuchen Cai, Xiaofeng Zhai, Jie Liu, Wen Zhao, Senlin Ji, Shuo Su, Jiyong Zhou

**Affiliations:** 1 MOE International Joint Collaborative Research Laboratory for Animal Health & Food Safety, Institute of Immunology and College of Veterinary Medicine, Nanjing Agricultural University, Nanjing 210095, China; 2017207021@njau.com (X.Z.); caiyuchen1114@gmail.com (Y.C.); 18851175127@163.com (X.Z.); 2016107063@njau.edu.cn (J.L.); 2017207031@njau.edu.cn (W.Z.); senlinji@163.com (S.J.); 2 Key Laboratory of Animal Virology of Ministry of Agriculture, Department of Veterinary Medicine, Zhejiang University, Hangzhou 310058, China

**Keywords:** RABV, lyssaviruses, codon usage bias, natural selection

## Abstract

Rabies virus (RABV) and other lyssaviruses can cause rabies and rabies-like diseases, which are a persistent public health threat to humans and other mammals. Lyssaviruses exhibit distinct characteristics in terms of geographical distribution and host specificity, indicative of a long-standing diversification to adapt to the environment. However, the evolutionary diversity of lyssaviruses, in terms of codon usage, is still unclear. We found that RABV has the lowest codon usage bias among lyssaviruses strains, evidenced by its high mean effective number of codons (ENC) (53.84 ± 0.35). Moreover, natural selection is the driving force in shaping the codon usage pattern of these strains. In summary, our study sheds light on the codon usage patterns of lyssaviruses, which can aid in the development of control strategies and experimental research.

## 1. Introduction

Biologists are devoted to exploring the complexity of evolutionary interactions among divergent viruses and their underlying reservoirs, and apply latent theoretical tenets to resolve practical cases. Viruses from the genus *Lyssavirus*, usually called lyssaviruses, belonging to *Rhabdoviridae* of the *Mononegavirales* order, present a classical case to study the emergence and cross-species transmission of infectious disease [1]. Rabies is an acute and almost invariably fatal encephalomyelitis in humans, usually caused by rabies virus (RABV) infection, which is a single-stranded, negative-sense, non-segmented RNA virus of approximately 12 kilo bases. The genome mainly encodes five proteins: The nucleoprotein (N), phosphoprotein (P), matrix protein (M), glycoprotein (G) and the large protein (L) [2,3]. RABV can infect a variety of mammalian hosts, especially bats and certain carnivores. It is distributed worldwide and has a high mortality, and remains a permanent threat to public health [4,5,6]; nevertheless, it is still neglected. Lyssaviruses are mainly classified into 16 species and, currently, two more tentative species. RABV, Lagos bat virus (LBV), Mokola virus (MOKV), Duvenhage virus (DUVV), European bat lyssavirus 1 (EBLV-1), European bat lyssavirus 2 (EBLV-2) and Australian bat lyssavirus (ABLV) are seven species that have been already identified. Other species have been recently identified, including Aravan virus (ARAV), Bokeloh bat lyssavirus (BBLV), Ikoma lyssavirus (IKOV), Irkut virus (IRKV), Khujand virus (KHUV), Shimoni bat virus (SHIBV), West Caucasian bat virus (WCBV), Lleida bat lyssavirus (LLEBV) and Gannoruwa bat lyssavirus (GBLV) [7,8,9,10]. Putative species, including Taiwan bat lyssavirus (TBLV) and Kotalahti bat lyssavirus (KBLV), have not yet been classified [11,12]. Historically, each species of lyssavirus is associated with a specific geographical area and is detected in different hosts and vectors [13]. For instance, EBLV-1 and EBLV-2 are found in serotine bats and Daubenton’s bats, respectively, in the United Kingdom, the Netherlands, Switzerland, and Norway, while ABLV is found in pteropid and insectivorous bats in Australia. In addition, RABV is distributed worldwide in dogs and several carnivores, except in Antarctica and a few islands, though it is commonly found in China and India [14,15,16]. However, the evolutionary relationship among these different viruses, caused by geographical isolation, is still not clear.

The genetic code is degenerated, meaning that an amino acid can be encoded by more than one codon. Codon usage is unbalanced in prokaryotes, eukaryotes, and viruses [17]. The preferential usage of codons is referred to as codon usage bias and is a widespread phenomenon in nature [18,19]. Mutation pressure and natural selection are the two main forces influencing codon usage patterns. Other factors include dinucleotide abundance, tRNA abundance, GC content, gene function, gene length, RNA structure, replication, and external environment, among others [20,21,22,23]. In terms of virus infection, the codon usage pattern of the respective host might affect virus survival, adaptation, evolution, and control of the host immune system, given that the virus relies on host cell machinery [24]. Thus, the study of codon usage patterns can provide more detailed information regarding virus evolution and a more detailed understanding of the pathogenesis, which can aid the development of drug targets for more effective vaccines and reinforce control measures to prevent the spread of this severe zoonosis.

RNA viruses have a high evolutionary rate; however, the evolution of lyssaviruses is relatively conserved [25]. Previous studies have mainly focused on pathogenesis or evolution to find amino acid sites under selection [26,27,28]. However, the evolutionary diversity among lyssaviruses, in terms of genome codon usage, is still unclear. In this study, we performed a large-scale and comprehensive codon usage analysis of lyssaviruses strains and determined the driving forces that influence the pattern of codon usage.

## 2. Results

### 2.1. Nucleotide Composition

Nucleotide composition constraints can influence the pattern of codon usage so we analyzed the composition of RABV coding sequences. The highest mean compositions of nucleotides were A (28.34 ± 0.15%) followed by U (26.03 ± 0.23%), C (22.93 ± 0.20%), and G (22.70 ± 0.16%). The mean nucleotides at the third positions of synonymous codons A_3s_ (32.69 ± 0.63%) and U_3s_ (31.58 ± 0.76%) were also higher than G_3s_ (31.31 ± 0.68%) and C_3s_ (30.33 ± 0.62%). The mean compositions of AU (54.37 ± 0.27%) were more than the GC compositions (45.63 ± 0.27%). Therefore, the AU content was higher than the GC in RABV. The same result was observed in the other lyssaviruses species, except for in EBLV-1, for which the contents of the third codon positions were G_3s_ 34.43 ± 0.27%, A_3s_ 31.41 ± 0.25%, U_3s_ 31.23 ± 0.32% and C_3s_ 29.65 ± 0.28%. These results indicated that the coding sequences of lyssaviruses are AU-rich (Table S1).

### 2.2. Codon Usage Bias Analysis

**Figure 1:**
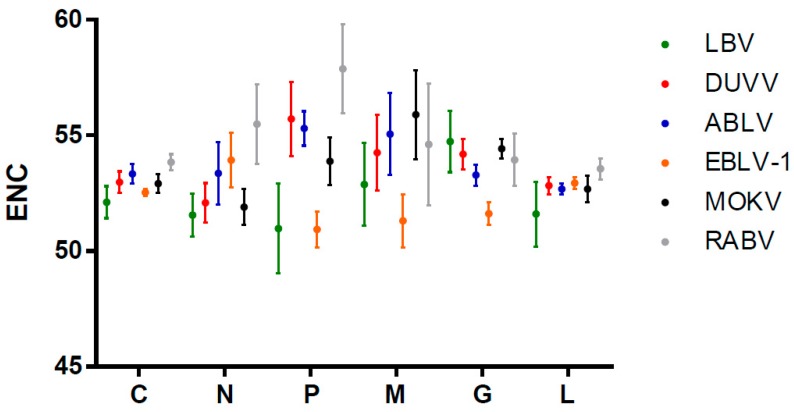
Species-specific comparative analysis of ENC (effective number of codons) values of each lyssavirus coding sequences. C means complete genome. Green, red, blue, orange, black and grey represent LBV (Lagos bat virus), DUVV (Duvenhage virus), ABLV (Australian bat lyssavirus), EBLV-1 (European bat lyssavirus 1), MOKV (Mokola virus) and RABV (rabies virus), respectively.

The ENC (effective number of codons) values were calculated to infer the degree of the codon usage bias of lyssaviruses. ENC values were calculated using complete coding sequences of different lyssavirus species strains and were then compared to identify differences among these strains. We found a low codon usage bias with the highest mean ENC value for RABV (53.84 ± 0.35) and the lowest mean ENC value for LBV (52.11 ± 0.69). Then, we calculated the mean ENC value of individual genes from different lyssaviruses strains (Figure 1). More sequences were added for this analysis, as described in Table S2. The highest ENC value corresponded to the P gene of RABV (57.87 ± 1.93). For DUVV and ABLV, the highest ENC values also corresponded to the P gene. Regarding other genes, the ENC values of other lyssavirus strains were different from that of RABV. These observed results suggested that, during the evolution of lyssaviruses, codon usage is relatively conserved and species-specific.

### 2.3. Codon Usage Indices Analysis

To reveal the pattern of synonymous codons of RABV and other lyssaviruses, we performed RSCU (relative synonymous codon usage) analysis of the 59 codons. In RABV, among the 18 most used synonymous codons, 10 were G-and C-ended (6 G-ended; 4 C-ended) and the other 8 were A- and U-ended (6 U-ended; 2 A-ended), so the preferentially used codons were G- and U-ended codons. However, for other lyssaviruses the preferred codons were A- and U-ended (LBV: 6 A-ended and 8 U-ended; MOKV: 7 A-ended and 5 U-ended; DUVV: 5 A-ended and 5 U-ended; ABLV: 4 A-ended and 7 U-ended). Interestingly, the preferred codons of EBLV were equally ended in A- and U- or G- and C-ended (2 A-ended, 7 U-ended, 3 C-ended and 6 G-ended). Next, we found that 2 of the 18 preferred codons in RABV (UCU for Ser and AGA for Arg) had RSCU values >1.6, and the remaining preferred codons had RSCU values >0.6 and <1.6. The number of over-represented codons of ABLV, EBLV and DUVV were same to RABV, MOKV and LBV had 3 preferred codons with RSCU values >1.6 (Table 1). None of the preferred codons were under-represented (RSCU < 0.6), regardless of the virus strain. Overall, the patterns of synonymous codons of RABV and other lyssaviruses are similar, though there are some differences in terms of preferred codons at the third position of synonymous codons.

### 2.4. Trends in Codon Usage Variations

**Figure 2:**
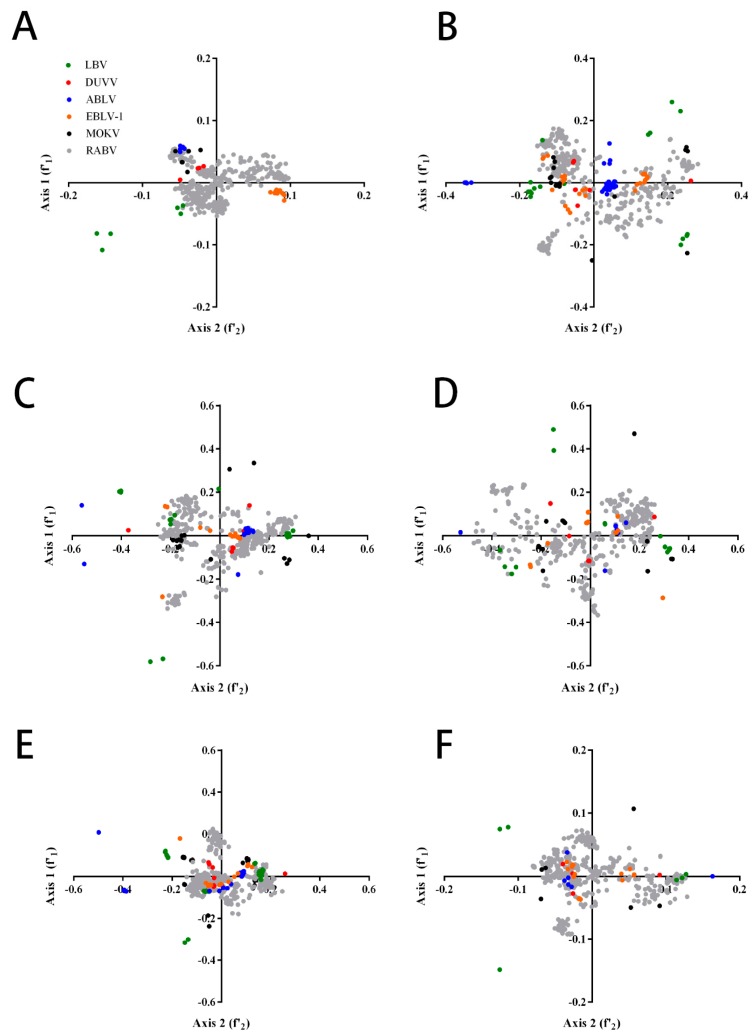
Principal component analysis. Species-specific PCA plots were constructed for whole genome and individual lyssavirus coding sequences. (**A**) Whole genome; (**B**) N; (**C**) P; (**D**) M; (**E**) G; (**F**) L. The color coding is the same as that of (**A**).

To dissect the variations in the codon usage trends among different lyssaviruses, we carried out PCA (principal component analysis) with the RSCU values of the genome coding sequences and the individual coding sequences. The average of the first (f’1) and second (f’2) principal axes accounted for 26.1% and 12.5%, occupying 38.6% of the total variation in the codon usage of RABV. The third (f’3) and fourth (f’4) axes accounted for 8.4% and 6.6% of the total variation in the codon usage of RABV, respectively. The downward trends in axes values were consistent with RABV for other lyssaviruses, indicating that the f’1 axes accounted for most of the codon usage variation (Figure S1). The plot first (f’1) axes against second (f’2) axes showed that lyssaviruses are divided into six groups, although there was a degree of overlap, indicating that these lyssaviruses strains may have the same ancestor. PCA also revealed that whole genome coding sequences of lyssaviruses strains were frequently distributed along the first (f’1) and second (f’2) principal axes except for LBV, while the individual coding sequences of lyssaviruses strains were diffusely distributed (Figure 2).

### 2.5. Identification of the Forces Influencing Codon Usage Patterns

**Figure 3:**
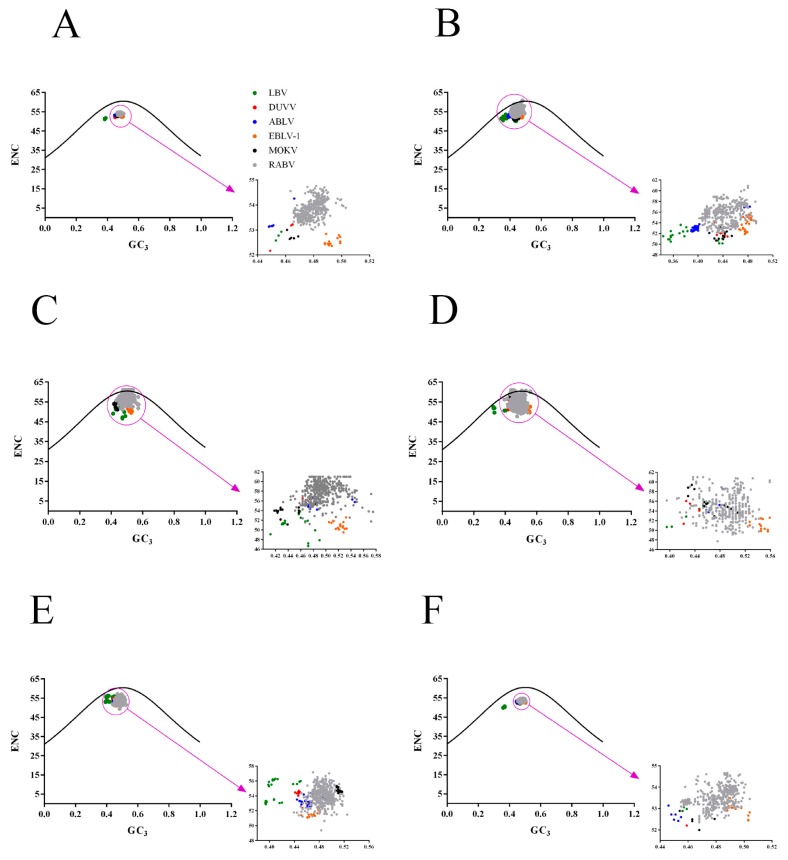
ENC-GC_3_ plot. (**A**) Whole genome; (**B**) N; (**C**) P; (**D**) M; (**E**) G; (**F**) L. The color coding is the same as that of (**A**).

**Figure 4:**
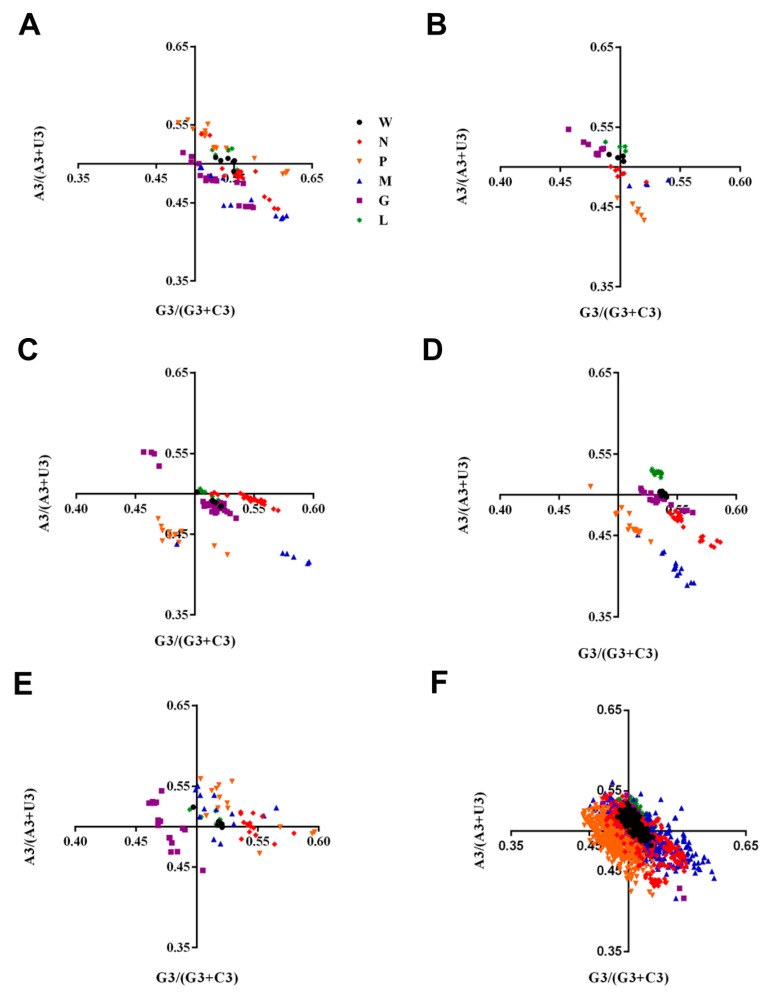
PR2 plot analysis. (**A**) LBV; (**B**) DUVV; (**C**) ABLV; (**D**) EBLV-1; (**E**) MOKV; (**F**) RABV. W means whole genome. Black, red, orange, blue, purple and green represent whole genome, N gene, P gene, M gene, G gene and L gene, respectively.

To establish the forces shaping the codon usage patterns of RABV and other lyssaviruses, we constructed ENC–GC_3s_ plots, PR2 (parity rule 2) bias, and correlations among the nucleotide compositions, codon compositions, Gravy, Aroma and principal axes. We found that the ENC values of all lyssaviruses strains occur below the expected ENC curve and clustered together except for LBV in ENC-GC_3s_ plots (Figure 3A), indicating that, except for mutation pressure, other factors, including natural selection, also drive the codon usage bias of RABV and other lyssaviruses strains. However, in the plot constructed using individual gene coding sequences, some points fell on the expected curve, for instance the N, P and M genes of RABV, the M gene of LBV and MOKV (Figure 3B–F). Interestingly, most of the LBV rarely clustered together with other lyssaviruses, regardless of the coding sequences of genome or individual genes (Figure 3), which is consistent with the plots of nucleotide distribution. To further analyze the impact of the highly biased genes restriction on codon choice, the relationships between the AU contents and the GC contents in the fourfold degenerate codon families (alanine, arginine, glycine, leucine, proline, serine, threonine and valine) were analyzed by PR2 plots (Figure 4). We found that the distribution of nucleotides was unequal in whole genome or individual gene coding sequences. Additionally, we discovered that in the four-codon amino acids family A ≠ U, G ≠ C, indicating that the driving forces are not sole and the extent of the influence is also not equal. We hypothesized that this may be due to a combination of mutation pressure and natural selection. Then we calculated the correlation of multiple factors. Several indices significantly correlated with the principal axes (Table 2 and Table S3), further confirming the above conclusion. Overall, natural selection and mutation pressure both have contributed to the codon usage bias of lyssaviruses strains.

### 2.6. Natural Selection Plays a Major Role in the Codon Usage Pattern of Lyssaviruses

**Figure 5:**
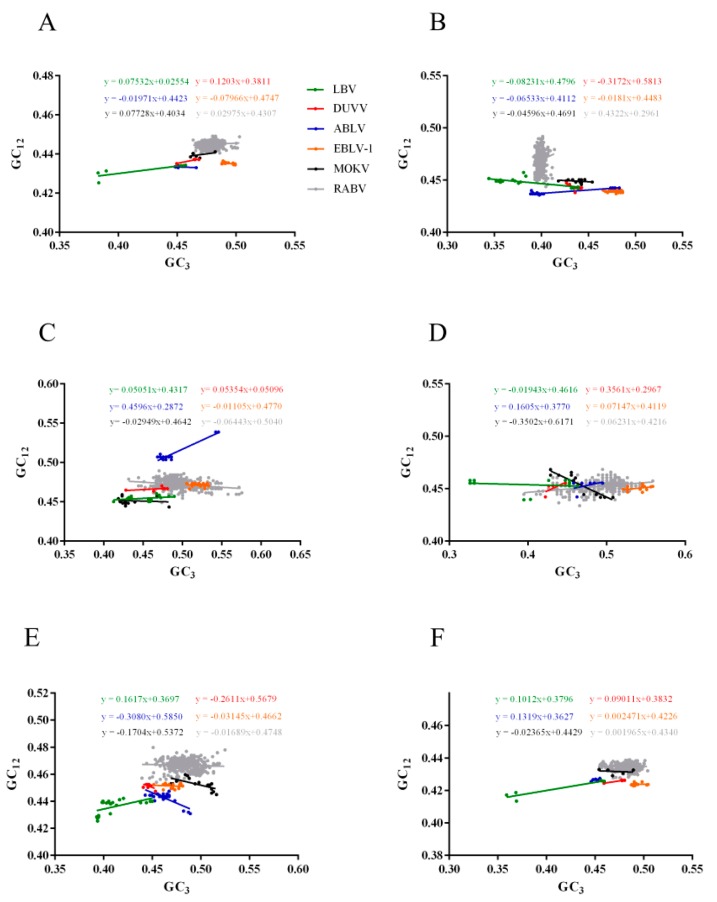
Neutrality plot analysis. (**A**) Whole genome; (**B**) N; (**C**) P; (**D**) M; (**E**) G; (**F**) L. The colour coding is the same as that of (**A**).

To determine the main factor shaping the codon usage pattern of the lyssaviruses, we performed neutrality plot analysis. We found a significant positive correlation between the P_12_ (GC_1,2s_) and P_3_ (GC_3s_) values (*p* = 0.003) of RABV. The P_12_ and P_3_ values of LBV (*p* = 0.042) and DUVV (*p* = 0.030) were positive correlated, whereas for EBLV (*p* = 0.011) there was a significant negative correlation between the P_12_ and P_3_ values. For MOKV (*p* = 0.342) and ABLV (*p* = 0.404) there was not a significantly correlation between the P_12_ and P_3_ values. Then, we calculated the slope of the regression line for each species lyssaviruses. The slope of RABV was 0.030 indicating that natural selection is the primary force influencing the codon usage patterns of RABV. The slopes of LBV, DUVV, EBLV-1, ABLV and MOKV were 0.075, 0.120, −0.080, −0.020 and 0.077 respectively. Thus, mutation pressures were 7.5%, 12.0%, 8.0% 2.0% and 7.7% and natural selection were 92.5%, 88%, 92%, 98% and 92.3%, respectively, demonstrating the dominant influence of natural selection in all lyssaviruses strains. Therefore, in comparison with mutation pressure, natural selection is the predominant force driving the codon usage of lyssaviruses (Figure 5).

### 2.7. Dinucleotide Abundance Influences the Codon Usage Bias of Lyssaviruses

We calculated the 16 dinucleotide abundance of lyssaviruses strains coding sequences to understand the possible effect in codon usage bias (Figure S2). We found that all the dinucleotide frequencies were not equal, and dinucleotides ApG, GpA and UpC were overrepresented, while dinucleotide CpG was underrepresented. Additionally, dinucleotide CpU was overrepresented in RABV and MOKV, while dinucleotides GpC and UpA were underrepresented in all the lyssaviruses strains coding sequences except for MOKV. Furthermore, the RSCU values of 8 CpG-containing codons (UCG, CCG, ACG, GCG, CAG, CGU, CGC, and CGG) were <1.6 indicating that dinucleotide CpG were inhibited. These results indicated dinucleotide abundance influences the codon usage bias of lyssaviruses.

## 3. Discussion

RABV, belong to the genus *Lyssavirus*, is the cause of acute zoonotic infectious diseases causing about 60,000 human deaths a year. Though the evolution of lyssaviruses, especially the RABV, has been previously investigated. However, many gaps still exist due to a lack of deep and systematic investigation. Here, we used 498 lyssaviruses sequences to perform a systematic and comprehensive analysis to understand the codon usage patterns during evolution and discriminate patterns of codon usage among different lyssaviruses species. The phenomenon of clustering among different lyssaviruses species in PCA plot demonstrates a significant correlation among these strains during evolution and that they may have diversified from a common ancestor as previously reported [29]. However, this still controversial [30], thus increased surveillance is needed to solve this dilemma.

In order to adapt to the changing of environment and the host, RNA viruses undergo evolutionary changes leading to genome divergence [31]. Codon usage bias is an important manifestation of gene evolution that can be influenced by many factors, the most common being natural selection and mutation pressure. We calculated ENC values and nucleotide composition and found that the highest mean ENC value was for RABV (53.84), indicating that the codon usage bias of RABV was the lowest. Previous studies have already reported low codon usage bias for RABV genes including, N [32] and G [33]. In addition, low codon usage bias has been identified in other RNA viruses, such as H5N1 influenza virus (50.91) [34], H3N8 Equine influenza virus (52.09) [35], Ebola virus (57.23) [36] and hepatitis C virus (HCV) (52.62) [37]. Low codon usage bias can help overcome host defense mechanisms and reduce the barriers for virus replication [38,39,40]. Therefore, it allows persistent infection in preferential host.

The analysis of nucleotide composition can reveal the use of preferred codons and reflect the effect of mutation pressure on codon usage bias. In lyssaviruses, the AU content was comparatively higher than the GC content in the overall genomic composition, demonstrating that codon usage bias plays a role in evolution. For RABV, despite the AU content being higher than the GC content, the preferred codons ended in G or U. However, for LBV, MOKV and DUVV, the majority of codons ended in A or U, consistent with the nucleotide content. Overall, this imbalance in codon usage can well account for the effect of mutation pressure on codon usage bias.

Moreover, we performed ENC–GC_3s_ plots, PR2 and correlation analysis to study the forces that drive codon usage bias. ENC-plot analysis showed that all strains of lyssaviruses occur below the expected ENC curve indicating that, except for mutation pressure, other factors including natural selection also drive the codon usage bias of RABV and other lyssaviruses strains. Additionally, most points in the plot constructed using individual gene coding sequences also occur below the expected ENC curve. In conclusion, mutation pressure is important in shaping the codon usage of lyssaviruses. Furthermore, the driving forces are not sole, and the effect of mutation pressure and natural selection is not equal revealed by PR2 analysis. In addition, the remarkable correlations between ENC, Gravy, Aroma and multiple factors revealed by correlation analysis indicated that natural selection contributes to the codon usage bias of lyssaviruses. We also constructed neutrality plots between the P_12_ and P_3_ values of complete genome and individual gene coding sequences and found that natural selection is the predominant force, consistent with a previous report [32].

Dinucleotide abundance is one factor influencing codon usage bias as previously described [35]. We found dinucleotides ApG, GpA and UpC were overrepresented in lyssaviruses, however dinucleotide CpG was underrepresented. And the un-methylated dinucleotide CpG can activate immune response by intracellular pattern recognition receptor-toll-like receptor 9 (TLR-9) [41,42]. Therefore, low CpG use is contributed to evading immune responses.

In summary, we performed a comprehensive analysis of the codon usage bias of six species viruses’ genome coding sequences from genus *Lyssavirus* from 1931 to present to further understand the evolution of lyssaviruses. Our results revealed that the codon usage bias of lyssaviruses is slight and that natural selection is a major factor influencing codon usage. Additionally, dinucleotide bias partly contributed to lyssaviruses codon usage patterns. Overall, these results will serve future lyssavirus surveillance and basic research.

## 4. Materials and Methods

### 4.1. Database

The coding sequences of 498 lyssaviruses genomes across different lineages reported worldwide between 1931 and 2016 were downloaded from the National Center for Biotechnological Information (http://www.ncbi.nlm.nih.gov/genbank/) (accessed on 29 October 2017) GenBank database. The detailed information regarding collection date, country, host and accession number is provided in Table S4. Different with many reported RNA viruses, which have a high rate on recombination [43,44,45], the rabies virus genome has rarely been reported previously [46], and so we excluded the effect of recombination on subsequent codon analysis in the screening of the database.

### 4.2. Nucleotide Composition Analysis

The codon compositions at the third position (A_3s_%, U_3s_%, C_3s_% and G_3s_%) were computed using Codon W 1.4.2. The frequencies of A, U, C and G (%) were calculated using Bio-edit. The GC content and GC_1s_, GC_2s_ and GC_3s_ were calculated using Emboss: cusp. The codon usage bias analysis excluded five codons including: AUG and UGG since they are the only codons encoding for Met and Trp, respectively and the termination codons UAA, UAG and UGA [39].

### 4.3. Relative Synonymous Codon Usage (RSCU) Analysis

RSCU indicates the relative probability of synonymous codons encoding an amino acid removing the effect of amino acid composition and coding sequence length. The RSCU index was calculated as follows:

(1) $$\mathrm{RSCU}\;=\;\frac{X_{ij}}{\sum_{j}^{n_{i}}X_{ij}}n_{i}$$

The observed number of the *i*_th_ codon for the *j*_th_ amino acid expressed as *X_ij_*, and *n_i_* is the number of synonymous codons that encode the *i*_th_ amino acid. A RSCU value >1.0 represents positive codon usage bias, while a RSCU value <1.0 indicates negative bias. A RSCU value of 1.0 indicates no codon usage bias [47]. Additionally, synonymous codons with RSCU values >1.6 and <0.6 indicate over-represented and under-represented codons respectively. RSCU values were calculated using MEGA (version 7.0) [48].

### 4.4. Principal Component (PCA) Analysis

PCA is a multivariate statistical method to analyze the relationship between variables and samples to identify major variation trends. PCA was used to identify clustering between the RSCU value of each strain using a 59-dimensional vector, excluding AUG, UGG and three termination codons [49]. PCA analysis was performed using the software Graphpad Prism 5.0 (GraphPad Software Inc., San Diego, CA, USA) against the classification based on different lyssaviruses [50].

### 4.5. Effective Number of Codons (ENC) Analysis

The ENC value describes the degree that the codon usage deviates from random selection and reflects the extent of preference for the non-equilibrium use of synonymous codons in the codon family. The values range from 20 to 61 [51]. The smaller the ENC value the stronger the bias [52]. The ENC value was calculated as follows:

(2) $$\mathrm{ENC}=2+\frac{9}{F_{2}}+\frac{1}{F_{3}}+\frac{5}{F_{4}}+\frac{3}{F_{6}}$$

where *F_i_* (*i* = 2, 3, 4, 6) represents the mean value of *F_i_* for *i*-fold degenerate codon families. The Fi value was calculated using the following formula:

(3) $$F_{i}=\frac{\sum_{j=1}^{i}{\left(\frac{n_{j}}{n}\right)}^{2}-1}{n-1}$$

*N* is the total number of occurrences of the codons for that amino acid and *n_j_* is the total number of frequencies of the *j*_th_ codon for that amino acid. In order to explore the factors influencing codon usage bias and to determine the relationship between the GC_3S_ and ENC values, the expected ENC was calculated as follows:

(4) $$ENC_{expected}=2+s+\frac{29}{s^{2}+\left(1-s^{2}\right)}$$

where ‘s’ is the frequency of G + C at the third codon position of synonymous codons. In ENC-GC_3s_ plots, if a point sits on the expected curve, it means mutation pressure is the only factor influencing evolution, whereas if it sits below the expected curve indicates that mutation pressure is not the sole evolutionary driving force [50].

### 4.6. Parity Rule 2 (PR2) Analysis

PR2 analysis, which explores the relationship between (A_3_/(A_3_ + U_3_) and (G_3_/(G_3_ + C_3_)) in the four-codon amino acids family, was used to demonstrate the effects of mutation pressure and natural selection on the codon usage of special genes. The points sitting in the center of the plot indicate A = U and G = C and therefore the effect of mutation and selection rates are equal [53,54].

### 4.7. Correlation Analysis

The correlations among the A%, U%, G%, C%, the codon on the third position (A_3_, U_3_, G_3_, C_3_ and GC_3_), GC_12_, ENC, Aroma, Gravy, Axis 1 and Axis 2 were calculated using GraphPad Prism (version 5.0). The correlation is determined by the *p* value. A *p* value < 0.01 means a strong significant correlation and 0.01 < *p* < 0.05 denotes significant correlation.

### 4.8. Neutrality Plot Analysis

Neutrality analysis was performed to identify the effects of natural selection and mutation pressure on the codon usage patterns by plotting the P_12_ (GC_1,2s_) values of the synonymous codons and the P_3_ (GC_3s_) values using Graphpad Prism 5.0 (GraphPad Software Inc., San Diego, CA, USA) [55]. The influence of natural selection and mutation pressure is expressed as the slope of a regression curve. If the slope of the regression curve is close to ±0.5, it indicates no or weak external selection pressure. When the slope is close to 0 or 1, it indicates a very low correlation between GC_1,2s_ and GC_3s_.

### 4.9. Dinucleotide Frequency Analysis

Dinucleotide frequency analysis was performed to estimate the dinucleotide abundances on codon usage patterns by using software DAMBE [56]. The frequencies of 16 dinucleotides were calculated as follows:

(5) $$P_{xy}=\frac{f_{xy}}{f_{x}f_{y}}$$

In the formula, *f_x_* and *f_y_* represent the frequency of nucleotide *X* and *Y*, respectively, while *f_xy_* represents the observed frequency of the dinucleotide *XY*, and *f_y_f_x_* represents the expected frequency of the dinucleotide value. It is considered that the *XY* dinucleotide is overrepresented and underrepresented when *P_xy_* > 1.23 and <0.78, respectively [57].

## Figures and Tables

**Table 1:** The relative synonymous codon usage (RSCU) patterns of lyssaviruses.

Amino Acid	Codon	RABV	ABLV	EBLV	LBV	DUVV	MOKV
Phe	UUU	0.95	**1.08**	0.86	**1.01**	0.87	0.93
	UUC	**1.05**	0.92	**1.14**	0.99	**1.13**	**1.07**
Leu	UUA	0.68	0.69	0.66	0.88	0.87	0.89
	UUG	**1.38**	**1.37**	**1.83**	**1.68**	**1.57**	**1.45**
	CUU	0.92	1.05	0.84	0.76	0.91	0.80
	CUC	0.89	0.93	0.88	0.71	0.96	0.88
	CUA	0.88	0.83	0.61	0.85	0.58	0.77
	CUG	1.25	1.13	1.18	1.12	1.12	1.22
Ile	AUU	0.80	0.89	0.83	1.02	0.96	0.82
	AUC	**1.16**	**1.06**	1.05	0.88	**1.03**	1.00
	AUA	1.04	1.04	**1.12**	**1.10**	1.01	**1.19**
Val	GUU	1.02	1.09	0.85	1.12	1.01	0.92
	GUC	1.15	**1.17**	1.03	0.87	**1.21**	1.16
	GUA	0.65	0.62	0.69	0.86	0.67	0.63
	GUG	**1.18**	1.12	**1.44**	**1.14**	1.11	**1.29**
Ser	UCU	**1.80**	**1.76**	**1.51**	**2.09**	**1.76**	**2.05**
	UCC	1.14	1.13	1.30	0.84	1.35	1.03
	UCA	1.38	1.43	1.37	1.42	1.37	1.24
	UCG	0.49	0.45	0.46	0.32	0.26	0.34
	AGU	0.64	0.69	0.76	0.78	0.80	0.64
	AGC	0.55	0.53	0.60	0.55	0.46	0.69
Pro	CCU	**1.49**	**1.48**	**1.58**	**1.57**	**1.43**	**1.61**
	CCC	1.07	0.81	0.83	0.89	0.95	1.01
	CCA	0.87	1.15	0.95	1.06	1.09	0.88
	CCG	0.56	0.55	0.64	0.48	0.53	0.50
Thr	ACU	1.13	1.22	**1.29**	1.18	1.08	1.29
	ACC	**1.32**	1.12	1.18	1.02	1.15	1.06
	ACA	1.21	**1.38**	1.16	**1.53**	**1.47**	**1.44**
	ACG	0.35	0.28	0.36	0.27	0.30	0.21
Ala	GCU	1.20	1.17	1.03	1.17	1.33	1.13
	GCC	1.12	1.18	1.25	0.96	0.95	1.07
	GCA	**1.31**	**1.37**	**1.43**	**1.55**	**1.38**	**1.42**
	GCG	0.37	0.27	0.29	0.32	0.34	0.38
Tyr	UAU	**1.11**	**1.16**	**1.14**	**1.36**	**1.11**	**1.12**
	UAC	0.89	0.84	0.86	0.64	0.89	0.88
His	CAU	**1.13**	**1.21**	**1.15**	**1.27**	0.99	**1.13**
	CAC	0.87	0.79	0.85	0.73	**1.01**	0.87
Gln	CAA	0.97	0.95	0.73	**1.09**	**1.06**	**1.06**
	CAG	**1.03**	**1.05**	**1.27**	0.91	0.94	0.94
Asn	AAU	0.89	0.90	0.94	**1.14**	0.86	0.91
	AAC	**1.11**	**1.10**	**1.06**	0.86	**1.14**	**1.09**
Lys	AAA	0.93	0.94	0.83	0.95	0.88	0.84
	AAG	**1.07**	**1.06**	**1.17**	**1.05**	**1.12**	**1.17**
Asp	GAU	**1.00**	**1.13**	**1.06**	**1.15**	**1.02**	**1.04**
	GAC	1.00	0.87	0.94	0.85	0.98	0.96
Glu	GAA	0.80	0.73	0.72	0.90	0.90	0.78
	GAG	**1.20**	**1.27**	**1.28**	**1.10**	**1.10**	**1.22**
Arg	CGU	0.23	0.36	0.17	0.24	0.27	0.42
	CGC	0.27	0.17	0.18	0.16	0.18	0.18
	CGA	0.63	0.52	0.47	0.70	0.60	0.55
	CGG	0.40	0.24	0.46	0.30	0.47	0.33
	AGA	**2.72**	**2.69**	**2.88**	**2.77**	**3.03**	**2.64**
	AGG	1.74	2.02	1.84	1.83	1.46	1.87
Gly	GGU	0.60	0.86	0.50	0.73	0.75	0.56
	GGC	0.59	0.52	0.55	0.47	0.41	0.53
	GGA	1.37	**1.47**	1.42	**1.49**	**1.43**	**1.49**
	GGG	**1.44**	1.15	1.53	1.31	1.42	1.42
Cys	UGU	**1.20**	**1.26**	**1.26**	**1.22**	**1.34**	**1.20**
	UGC	0.80	0.74	0.74	0.78	0.66	0.80

Preferred codons of each lyssavirus are shown in bold.

**Table 2:** Correlation analysis of RABV genome coding sequences.

	A%	C%	G%	U%	A3s	C3s	G3s	T3s	AU	GC	GC1s	GC2s	GC12s	ENC	Axis1	Axis2	Gravy
A%																	
C%	−0.060 ^NS^																
G%	−0.814 **	0.068 ^NS^															
U%	−0.018 ^NS^	−0.906 **	−0.243 **														
A3s	0.877 **	0.190 ^NS^	−0.750 **	−0.209 **													
C3s	−0.065 ^NS^	0.951 **	0.089 ^NS^	−0.874 **	0.124 **												
G3s	−0.738 **	−0.072 ^NS^	0.889 **	−0.090 ^NS^	−0.853 **	−0.009 ^NS^											
T3s	−0.040 ^NS^	−0.895 **	−0.200 **	0.973 **	−0.246 **	−0.910 **	−0.061 ^NS^										
AU	0.535 **	−0.793 **	−0.653 **	0.828 **	0.307 **	−0.769 **	−0.483 **	0.795 **									
GC	−0.535 **	0.793 **	0.653 **	−0.828 **	−0.307 **	0.769 **	0.483 **	−0.795 **	−1.000 **								
GC1s	0.020 ^NS^	0.496 **	−0.008	−0.451 **	0.345 **	0.282 **	−0.307 **	−0.342 **	−0.367 **	0.367 **							
GC2s	−0.312 **	0.308 **	0.353 **	−0.323 **	−0.002	0.171 **	0.018 ^NS^	−0.274 **	−0.437 **	0.437 **	0.289 **						
GC12s	−0.149 **	0.515 **	0.178 **	−0.491 **	0.246 **	0.291 **	−0.210 **	−0.388 **	−0.490 **	0.490 **	0.868 **	0.726 **					
ENC	−0.222 **	0.507 **	0.283 **	−0.513 **	−0.042 ^NS^	0.504 **	0.190 **	−0.528 **	−0.562 **	0.562 **	0.295 **	0.115 *	0.272 **				
Axis1	−0.521 **	0.105 *	0.686 **	−0.244 **	−0.555 **	0.173 **	0.679 **	−0.226 **	−0.494 **	0.494 **	−0.162 **	0.188 **	−0.018 ^NS^	0.170 **			
Axis2	−0.041 ^NS^	−0.644 **	0.121 **	0.518 **	−0.323 **	−0.632 **	0.197 **	0.594 **	0.417 **	−0.417 **	−0.238 **	−0.212 **	−0.282 **	−0.427 **	0.006 ^NS^		
Gravy	−0.030 ^NS^	−0.286 **	−0.004 ^NS^	0.280 **	−0.038 ^NS^	−0.297 **	−0.090 ^NS^	0.282 **	0.230 **	−0.230 **	0.116 *	−0.057 ^NS^	0.053 ^NS^	−0.254 **	0.053 ^NS^	0.430 **	
Aromo	−0.043 ^NS^	−0.104 *	−0.002 ^NS^	0.124 **	−0.071 ^NS^	−0.081 ^NS^	0.034 ^NS^	0.115 *	0.089 ^NS^	−0.089 ^NS^	−0.134 **	−0.019 ^NS^	−0.106 *	−0.140 **	−0.006 ^NS^	0.041 ^NS^	−0.162 **

Note: NS means non-significant (*p* > 0.05); * represents 0.01 < *p* < 0.05; ** represents *p* < 0.01.

## Supplementary Materials

Supplementary materials can be found at http://www.mdpi.com/1422-0067/19/8/2397/s1.
