# Supplementary material for: Comprehensive Analysis of Codon Usage on Rabies Virus and Other Lyssaviruses
Source: Int J Mol Sci. 2018 Aug 14;19(8):2397. doi: 10.3390/ijms19082397 (PMC6121662; doi:10.3390/ijms19082397)
Supplement: Supplementary file 1 [file ijms-19-02397-s001.zip › suppl fig.pdf]

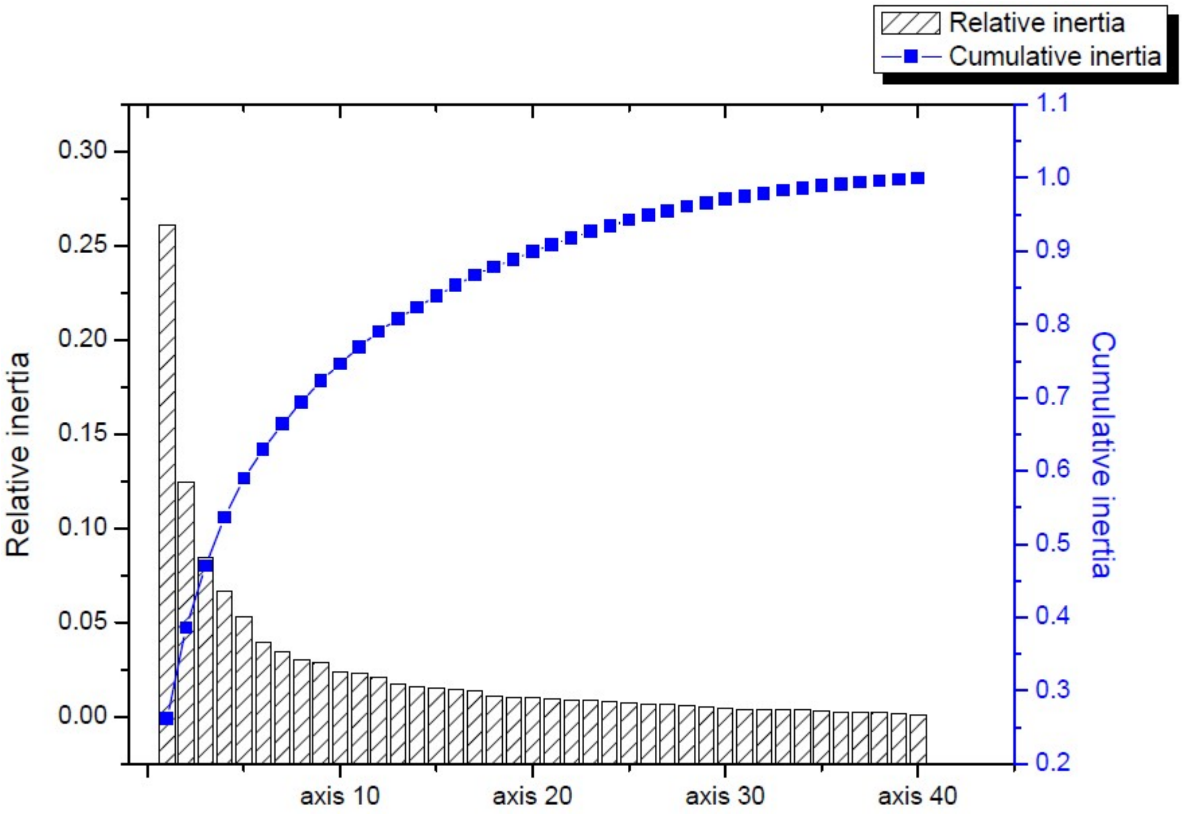

Figure S1. The relative and cumulative inertia of the first 40 axes accounting for 100% of the 59 axes.

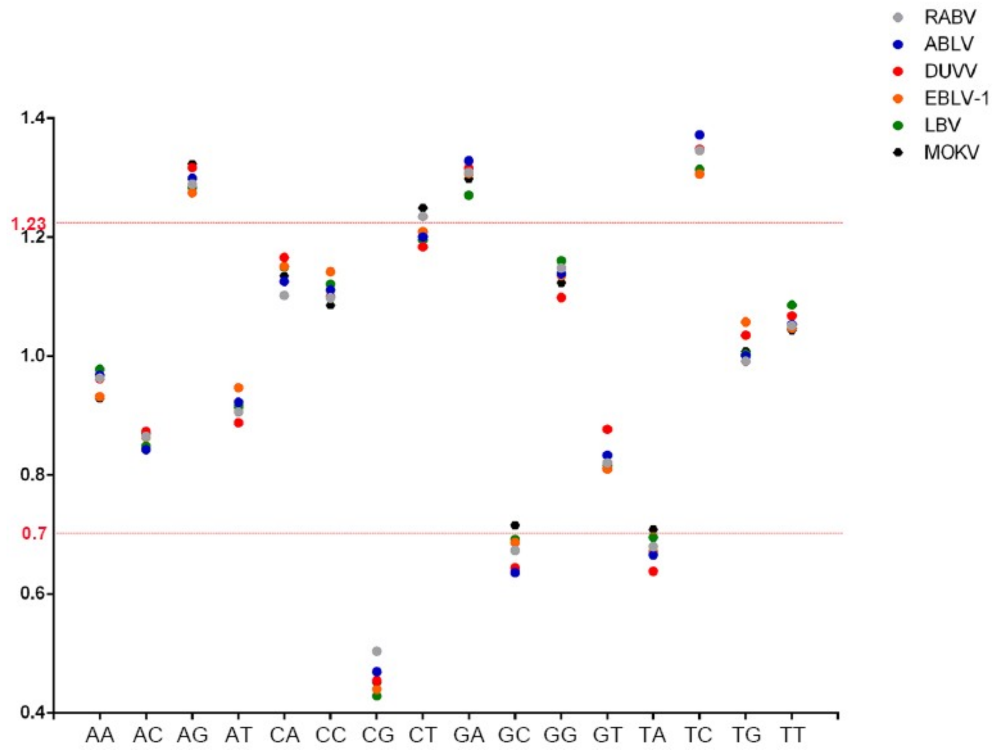

Figure S2. Dinucleotide frequency analysis. Table S1. Characteristic of lyssavirus genomes. Table S2. Nucleotide composition analysis.
