# Supplementary material for: Comprehensive Analysis of Codon Usage on Rabies Virus and Other Lyssaviruses
Source: Int J Mol Sci. 2018 Aug 14;19(8):2397. doi: 10.3390/ijms19082397 (PMC6121662; doi:10.3390/ijms19082397)
Supplement: Supplementary file 1 [file ijms-19-02397-s001.zip › Table S1 Nucleotide composition analysis .pdf]

Supplemental table 1 Nucleotide composition analysis of lyssavirus coding sequences (%).

A. Nucleotide composition analysis of RABV coding sequences (%).

No	A	T	G	C	A_3	T_3	G_3	C_3	AU	GC	GC_1	GC_2	GC_3	GC_{12}	ENC
1	28.41	26.31	22.54	22.73	32.58	31.99	31.31	30.12	54.70	45.30	48.53	39.63	47.66	44.08	54.13
2	28.29	26.34	22.61	22.76	32.40	31.86	31.36	30.36	54.60	45.40	48.56	39.63	47.93	44.10	53.84
3	28.41	26.11	22.60	22.89	32.97	31.91	30.58	30.28	54.50	45.50	49.28	39.77	47.39	44.52	54.47
4	28.31	26.54	22.64	22.51	32.30	33.24	31.04	29.19	54.90	45.10	48.81	39.90	46.73	44.35	53.61
5	28.22	26.05	22.74	22.99	32.59	31.52	31.23	30.51	54.30	45.70	49.29	39.86	48.05	44.57	54.01
6	28.24	26.12	22.74	22.90	31.68	31.81	31.90	30.72	54.40	45.60	48.83	39.55	48.54	44.19	53.74
7	28.62	25.92	22.39	23.08	34.27	30.97	29.61	30.74	54.50	45.50	49.48	39.76	47.16	44.62	53.49
8	28.64	25.92	22.37	23.08	34.27	31.02	29.58	30.75	54.60	45.40	49.45	39.76	47.14	44.60	53.50
9	28.64	25.93	22.36	23.08	34.31	31.01	29.54	30.74	54.60	45.40	49.45	39.76	47.11	44.60	53.50
10	28.62	25.92	22.37	23.10	34.35	30.96	29.50	30.76	54.50	45.50	49.51	39.79	47.11	44.65	53.50
11	28.61	25.93	22.38	23.09	34.27	31.01	29.58	30.74	54.50	45.50	49.51	39.76	47.14	44.63	53.53
12	28.34	26.45	22.63	22.58	32.10	33.05	31.74	29.30	54.80	45.20	48.77	39.63	47.23	44.20	53.39
13	28.33	26.48	22.63	22.56	32.30	32.98	31.56	29.34	54.80	45.20	48.68	39.75	47.14	44.22	53.50
14	28.35	26.21	22.71	22.72	32.15	32.23	31.87	29.91	54.60	45.40	48.78	39.64	47.88	44.21	53.57
15	28.40	26.20	22.68	22.71	32.14	32.36	31.82	29.90	54.60	45.40	48.78	39.61	47.79	44.20	53.41
16	28.33	26.39	22.61	22.67	32.24	32.82	31.61	29.55	54.70	45.30	48.93	39.59	47.33	44.26	53.63
17	28.10	26.12	22.94	22.83	31.79	32.06	32.05	30.02	54.20	45.80	49.39	39.75	48.18	44.57	54.76
18	28.43	26.41	22.62	22.54	32.50	33.00	31.59	29.02	54.80	45.20	48.94	39.58	46.94	44.26	53.60
19	28.36	26.03	22.51	23.10	33.07	31.49	30.62	30.64	54.40	45.60	49.32	39.81	47.70	44.56	53.58
20	28.40	26.36	22.61	22.62	32.39	32.66	31.73	29.43	54.80	45.20	48.72	39.65	47.33	44.19	53.28
21	28.32	26.38	22.64	22.63	32.01	32.83	31.83	29.53	54.70	45.30	48.74	39.61	47.49	44.17	53.44
22	28.36	26.48	22.63	22.53	32.20	33.27	31.87	28.94	54.80	45.20	48.98	39.50	46.98	44.24	53.34

23	28.27	25.94	22.90	22.89	32.47	31.18	31.81	30.40	54.20	45.80	48.98	40.05	48.35	44.52	53.85
24	28.16	25.80	23.03	23.00	32.08	31.08	32.28	30.47	54.00	46.00	49.32	40.04	48.74	44.68	53.84
25	28.64	25.92	22.37	23.08	33.50	30.85	30.60	31.09	54.60	45.40	48.91	39.47	47.96	44.19	53.01
26	28.09	26.20	23.00	22.71	31.49	32.45	32.95	29.07	54.30	45.70	49.02	40.13	47.97	44.57	53.39
27	28.27	25.95	22.88	22.90	32.51	31.21	31.77	30.36	54.20	45.80	49.01	40.05	48.29	44.53	53.83
28	28.21	25.77	22.98	23.04	32.18	30.88	32.20	30.68	54.00	46.00	49.25	39.97	48.84	44.61	53.78
29	28.20	25.95	22.95	22.91	32.22	31.22	31.99	30.44	54.10	45.90	49.00	40.06	48.51	44.53	53.89
30	28.30	26.10	22.63	22.97	32.77	31.68	30.92	30.49	54.40	45.60	49.20	39.80	47.78	44.50	54.17
31	28.01	26.05	23.14	22.80	31.02	31.90	33.17	29.83	54.10	45.90	48.99	40.03	48.78	44.51	53.22
32	27.99	26.08	23.10	22.82	31.19	31.91	32.96	29.74	54.10	45.90	48.97	40.17	48.62	44.57	53.42
33	28.16	26.04	22.94	22.86	31.86	31.43	32.41	30.31	54.20	45.80	49.04	39.68	48.67	44.36	53.95
34	28.15	25.79	23.04	23.01	32.08	31.04	32.28	30.50	53.90	46.10	49.35	40.04	48.77	44.69	53.85
35	28.34	26.57	22.57	22.52	32.34	33.48	31.22	28.96	54.90	45.10	49.23	39.44	46.60	44.34	53.50
36	28.11	25.79	23.07	23.02	31.97	31.03	32.39	30.49	53.90	46.10	49.35	40.07	48.85	44.71	53.79
37	28.22	25.91	22.92	22.96	32.22	31.12	31.96	30.58	54.10	45.90	48.97	40.06	48.59	44.52	53.84
38	28.13	25.83	23.00	23.04	31.99	31.01	32.13	30.70	54.00	46.00	49.14	40.12	48.85	44.63	53.67
39	28.17	26.08	23.01	22.74	31.70	31.82	32.72	29.68	54.20	45.80	48.93	39.98	48.35	44.45	53.19
40	28.19	25.79	23.00	23.02	32.16	30.98	32.25	30.53	54.00	46.00	49.26	40.04	48.77	44.65	53.83
41	28.27	25.95	22.90	22.88	32.51	31.21	31.77	30.36	54.20	45.80	49.01	40.05	48.29	44.53	53.85
42	28.15	25.91	23.00	22.95	32.07	31.19	32.10	30.51	54.10	45.90	49.12	40.06	48.65	44.59	53.79
43	28.20	25.97	22.96	22.88	32.02	31.29	32.41	30.31	54.20	45.80	49.10	39.75	48.67	44.42	53.91
44	28.10	26.15	22.87	22.87	31.35	31.98	32.41	30.45	54.30	45.70	49.16	39.47	48.61	44.32	54.02
45	28.36	26.24	22.43	22.97	32.27	32.37	30.70	30.56	54.60	45.40	49.07	39.59	47.54	44.33	53.83
46	28.23	25.87	23.00	22.89	32.24	31.27	32.26	30.18	54.10	45.90	49.28	39.94	48.46	44.61	53.64
47	28.52	26.09	22.63	22.75	32.77	31.84	31.37	30.06	54.60	45.40	48.71	39.81	47.64	44.26	53.80

48	28.03	25.85	23.09	23.03	31.35	31.31	32.98	30.19	53.90	46.10	49.19	40.16	49.02	44.67	53.81
49	28.19	26.12	22.89	22.80	31.51	32.02	32.24	30.31	54.30	45.70	49.06	39.55	48.48	44.31	53.82
50	28.06	26.60	23.03	22.31	31.56	33.88	32.03	28.49	54.70	45.30	49.27	39.94	46.81	44.61	53.99
51	28.22	26.19	22.85	22.75	32.06	32.60	32.04	29.14	54.40	45.60	49.19	40.18	47.42	44.68	53.52
52	28.15	25.78	23.04	23.03	32.04	30.98	32.32	30.57	53.90	46.10	49.32	40.04	48.85	44.68	53.85
53	28.01	26.26	23.09	22.63	31.24	32.37	32.79	29.58	54.30	45.70	49.16	39.69	48.32	44.42	53.71
54	28.16	25.79	23.03	23.02	32.13	30.98	32.20	30.57	54.00	46.00	49.27	40.08	48.78	44.68	53.95
55	28.17	25.78	23.02	23.03	32.07	31.06	32.25	30.52	53.90	46.10	49.32	40.07	48.77	44.69	53.88
56	28.27	25.95	22.90	22.88	32.47	31.21	31.81	30.36	54.20	45.80	48.98	40.05	48.32	44.52	53.85
57	28.44	25.93	22.44	23.19	32.43	31.69	30.65	31.08	54.40	45.60	49.06	39.81	48.02	44.44	53.30
58	28.19	25.78	23.01	23.02	32.12	30.98	32.29	30.53	54.00	46.00	49.26	40.04	48.80	44.65	53.83
59	28.23	25.89	22.92	22.96	32.02	31.03	32.36	30.63	54.10	45.90	49.06	39.70	48.88	44.38	53.80
60	28.16	25.80	23.03	23.00	32.04	31.08	32.33	30.47	54.00	46.00	49.29	40.04	48.77	44.67	53.84
61	28.18	25.81	22.98	23.03	32.02	31.04	32.11	30.64	54.00	46.00	49.14	40.09	48.80	44.62	53.61
62	28.28	25.96	22.89	22.87	32.47	31.22	31.81	30.37	54.20	45.80	48.98	39.99	48.32	44.49	53.86
63	28.09	26.32	22.87	22.72	31.60	32.62	31.82	29.73	54.40	45.60	49.12	39.86	47.79	44.49	53.86
64	28.25	25.95	22.92	22.88	32.43	31.21	31.85	30.36	54.20	45.80	49.01	40.05	48.35	44.53	53.86
65	28.26	26.25	22.57	22.92	31.74	32.17	31.40	30.74	54.50	45.50	48.91	39.34	48.22	44.13	54.12
66	28.26	25.95	22.91	22.88	32.47	31.21	31.81	30.36	54.20	45.80	49.01	40.05	48.32	44.53	53.86
67	28.18	25.80	23.01	23.00	32.11	30.97	32.33	30.52	54.00	46.00	49.25	40.00	48.81	44.62	53.86
68	28.18	25.93	22.96	22.94	32.22	31.18	31.98	30.47	54.10	45.90	49.09	40.06	48.53	44.57	53.77
69	28.29	26.03	22.81	22.87	32.42	31.27	31.76	30.42	54.30	45.70	48.89	39.82	48.33	44.35	53.44
70	28.38	26.11	22.50	23.01	33.11	31.54	30.53	30.76	54.50	45.50	49.35	39.49	47.69	44.42	53.89
71	28.43	25.81	22.56	23.20	33.02	30.86	30.72	31.30	54.20	45.80	49.15	39.84	48.30	44.49	53.50
72	28.18	25.76	22.99	23.07	32.07	30.89	32.28	30.72	53.90	46.10	49.30	39.94	48.93	44.62	53.84

73	28.19	25.88	22.96	22.97	32.12	31.08	32.06	30.64	54.10	45.90	49.04	40.02	48.72	44.53	53.61
74	28.15	25.89	22.99	22.98	31.62	31.45	32.74	30.06	54.00	46.00	49.12	40.08	48.71	44.60	53.29
75	28.19	25.97	22.97	22.88	31.98	31.29	32.46	30.31	54.20	45.80	49.10	39.75	48.70	44.42	53.91
76	28.15	25.79	23.04	23.01	32.01	31.04	32.37	30.50	53.90	46.10	49.29	40.04	48.83	44.67	53.83
77	28.23	25.86	22.95	22.97	32.31	31.21	32.03	30.36	54.10	45.90	49.30	39.97	48.46	44.64	53.86
78	28.19	25.85	22.96	23.00	32.21	31.11	31.94	30.57	54.00	46.00	49.19	40.08	48.61	44.63	53.68
79	28.34	26.30	22.67	22.69	32.56	32.29	31.20	29.92	54.60	45.40	49.19	39.49	47.39	44.34	53.52
80	27.96	26.61	22.92	22.51	31.58	33.18	31.79	29.07	54.60	45.40	48.87	40.19	47.25	44.53	54.02
81	28.45	25.88	22.45	23.23	32.42	31.33	30.81	31.36	54.30	45.70	48.91	39.79	48.33	44.35	53.73
82	28.23	26.19	22.76	22.81	32.24	32.05	31.56	30.04	54.40	45.60	48.96	39.94	47.83	44.45	54.04
83	28.21	25.78	22.97	23.04	32.13	30.97	32.21	30.63	54.00	46.00	49.25	39.97	48.81	44.61	53.77
84	28.31	26.03	22.72	22.94	32.61	31.79	31.29	30.19	54.30	45.70	49.38	39.84	47.78	44.61	53.79
85	28.16	25.79	23.03	23.02	32.08	31.01	32.28	30.53	53.90	46.10	49.32	40.04	48.80	44.68	53.84
86	28.17	25.79	23.02	23.01	32.08	30.94	32.33	30.57	54.00	46.00	49.23	40.01	48.85	44.62	53.86
87	28.23	25.74	22.98	23.05	32.26	30.82	32.21	30.65	54.00	46.00	49.19	40.05	48.84	44.62	53.74
88	28.25	25.78	22.93	23.04	32.33	31.05	31.99	30.54	54.00	46.00	49.36	39.97	48.58	44.67	53.69
89	28.18	25.80	23.00	23.01	32.08	30.98	32.29	30.57	54.00	46.00	49.20	40.01	48.83	44.61	53.83
90	28.34	26.46	22.74	22.45	32.58	32.92	31.36	29.11	54.80	45.20	49.28	39.45	46.87	44.36	53.37
91	28.19	25.91	22.96	22.95	32.22	31.17	31.98	30.52	54.10	45.90	49.09	40.06	48.56	44.57	53.73
92	28.47	26.28	22.61	22.64	32.86	32.47	31.09	29.59	54.70	45.30	49.22	39.52	47.02	44.37	53.40
93	28.15	25.80	23.04	23.00	32.01	31.08	32.37	30.47	54.00	46.00	49.29	40.04	48.80	44.67	53.84
94	28.24	26.31	22.55	22.90	31.57	32.39	31.47	30.68	54.50	45.50	48.91	39.25	48.19	44.08	53.91
95	28.37	26.04	22.55	23.04	33.02	31.45	30.35	31.01	54.40	45.60	49.30	39.68	47.80	44.49	53.52
96	28.16	26.49	22.89	22.46	31.68	32.94	32.22	29.16	54.70	45.30	48.91	39.61	47.52	44.26	53.47
97	28.26	25.97	22.91	22.86	32.47	31.28	31.81	30.30	54.20	45.80	49.01	40.05	48.26	44.53	53.84

98	28.19	25.95	22.97	22.89	31.98	31.23	32.46	30.38	54.10	45.90	49.10	39.75	48.75	44.42	53.90
99	28.21	25.93	22.92	22.95	32.26	31.22	31.85	30.51	54.10	45.90	49.06	40.06	48.48	44.56	53.87
100	28.20	25.88	22.67	23.25	32.44	31.02	31.30	31.26	54.10	45.90	49.42	39.68	48.64	44.55	54.45
101	28.26	25.94	22.91	22.89	32.43	31.18	31.85	30.40	54.20	45.80	48.98	40.05	48.38	44.52	53.87
102	28.44	26.38	22.57	22.60	33.32	32.54	30.83	29.16	54.80	45.20	49.04	39.90	46.58	44.47	53.78
103	28.17	26.06	22.84	22.93	32.29	31.96	31.54	29.96	54.20	45.80	49.38	40.10	47.84	44.74	53.96
104	28.45	26.10	22.65	22.80	33.48	31.74	30.69	29.90	54.60	45.40	49.29	39.91	47.15	44.60	54.02
105	28.45	26.09	22.58	22.88	32.75	31.97	30.84	30.24	54.50	45.50	49.20	39.68	47.50	44.44	53.46
106	28.47	26.02	22.54	22.97	33.21	31.82	30.64	30.25	54.50	45.50	49.32	39.87	47.35	44.60	53.45
107	28.37	26.41	22.62	22.61	32.17	32.82	31.74	29.49	54.80	45.20	48.68	39.61	47.38	44.14	53.56
108	28.35	26.46	22.62	22.57	32.16	33.02	31.76	29.27	54.80	45.20	48.72	39.62	47.22	44.17	53.58
109	28.38	26.37	22.62	22.64	32.22	32.73	31.75	29.56	54.70	45.30	48.80	39.55	47.43	44.17	53.57
110	28.52	26.51	22.53	22.44	32.85	33.14	31.28	28.92	55.00	45.00	48.75	39.59	46.58	44.17	53.33
111	28.54	26.20	22.55	22.71	32.83	32.34	31.45	29.65	54.70	45.30	48.98	39.50	47.30	44.24	53.27
112	28.39	26.52	22.61	22.49	32.21	33.17	31.75	29.12	54.90	45.10	48.68	39.55	47.06	44.11	53.46
113	28.35	26.35	22.65	22.64	32.27	32.69	31.82	29.45	54.70	45.30	48.87	39.59	47.42	44.23	53.37
114	28.35	25.84	22.66	23.15	33.15	30.77	30.95	30.94	54.20	45.80	49.19	40.01	48.23	44.60	54.01
115	28.37	25.83	22.66	23.14	33.22	30.73	30.95	30.90	54.20	45.80	49.19	40.01	48.20	44.60	54.03
116	28.45	25.90	22.62	23.03	33.40	30.88	30.92	30.64	54.30	45.70	48.99	40.03	47.94	44.51	53.98
117	28.42	25.89	22.64	23.06	33.36	30.80	30.96	30.73	54.30	45.70	49.03	40.02	48.04	44.52	54.02
118	28.41	26.32	22.68	22.59	32.64	32.73	31.58	29.32	54.70	45.30	49.13	39.55	47.13	44.34	53.31
119	28.40	26.04	22.58	22.98	33.11	31.85	30.40	30.36	54.40	45.60	49.48	39.87	47.34	44.67	53.92
120	28.45	26.17	22.58	22.80	33.10	32.29	30.66	29.78	54.60	45.40	49.41	39.74	46.98	44.57	53.78
121	28.38	26.45	22.61	22.57	32.21	32.99	31.69	29.32	54.80	45.20	48.71	39.61	47.20	44.16	53.46
122	28.38	25.71	22.89	23.02	32.82	30.59	32.17	30.39	54.10	45.90	49.20	39.93	48.59	44.57	54.57

123	28.21	26.29	22.81	22.70	32.71	32.42	31.46	29.16	54.50	45.50	49.38	40.02	47.12	44.70	53.66
124	28.34	26.44	22.62	22.61	32.06	33.02	31.74	29.37	54.80	45.20	48.77	39.61	47.29	44.19	53.52
125	28.15	25.87	22.80	23.17	32.37	31.40	31.54	30.48	54.00	46.00	49.62	40.00	48.29	44.81	54.45
126	28.23	25.86	22.80	23.12	32.67	31.35	31.30	30.40	54.10	45.90	49.62	40.06	48.06	44.84	54.08
127	28.24	25.80	22.79	23.16	32.78	31.29	31.17	30.44	54.00	46.00	49.71	40.12	48.00	44.92	54.02
128	28.25	25.73	22.81	23.22	32.71	31.07	31.24	30.63	54.00	46.00	49.72	40.11	48.25	44.92	54.02
129	28.42	26.07	22.38	23.13	33.21	31.63	30.00	30.95	54.50	45.50	49.22	39.80	47.51	44.51	53.83
130	28.53	26.41	22.60	22.46	32.73	32.79	31.75	29.14	54.90	45.10	48.77	39.37	47.03	44.07	53.57
131	28.36	26.34	22.67	22.63	32.29	32.65	31.85	29.39	54.70	45.30	48.85	39.66	47.41	44.26	53.36
132	28.27	26.16	22.82	22.75	32.43	32.19	31.42	29.74	54.40	45.60	49.35	39.82	47.55	44.58	54.72
133	28.30	26.28	22.67	22.76	32.91	32.13	31.10	29.69	54.60	45.40	49.04	39.98	47.25	44.51	53.99
134	28.36	25.78	22.67	23.19	33.03	30.90	31.10	30.90	54.10	45.90	49.42	39.92	48.23	44.67	54.40
135	28.31	26.37	22.64	22.68	32.42	32.14	31.55	29.86	54.70	45.30	48.58	39.73	47.65	44.16	54.13
136	28.62	25.94	22.47	22.97	33.90	31.23	30.28	30.41	54.60	45.40	49.13	39.90	47.28	44.51	53.77
137	28.45	26.07	22.51	22.97	33.36	31.69	30.37	30.37	54.50	45.50	49.22	39.91	47.30	44.57	53.66
138	28.24	25.80	22.80	23.16	32.38	30.84	31.74	31.04	54.00	46.00	49.15	39.90	48.83	44.52	54.23
139	28.00	26.15	22.98	22.87	30.81	31.88	33.20	30.39	54.20	45.80	48.80	39.58	49.17	44.19	53.57
140	28.04	26.13	23.02	22.78	31.00	32.01	33.24	30.03	54.20	45.80	48.84	39.68	48.90	44.26	53.70
141	28.44	26.30	22.63	22.63	32.84	32.45	31.56	29.23	54.70	45.30	48.77	39.89	47.11	44.33	53.85
142	28.37	25.73	22.84	23.06	32.74	30.65	31.92	30.68	54.10	45.90	49.26	39.81	48.62	44.54	54.62
143	28.40	26.37	22.57	22.65	32.40	32.71	31.50	29.54	54.80	45.20	48.71	39.66	47.29	44.19	53.62
144	28.30	26.46	22.71	22.53	32.00	33.13	31.90	29.25	54.80	45.20	48.87	39.57	47.28	44.22	53.44
145	28.29	26.17	22.73	22.80	32.86	32.17	31.14	29.56	54.50	45.50	49.35	40.05	47.21	44.70	53.51
146	28.42	26.32	22.48	22.79	33.20	32.96	30.35	29.32	54.70	45.30	49.80	39.64	46.35	44.72	54.56
147	28.10	25.53	22.93	23.44	31.00	30.57	32.87	31.52	53.60	46.40	48.97	40.13	50.01	44.55	53.99

148	27.68	25.64	23.32	23.37	30.49	30.68	33.44	31.32	53.30	46.70	49.74	40.02	50.29	44.88	53.89
149	28.21	26.19	22.77	22.83	31.62	32.15	32.81	29.84	54.40	45.60	48.97	39.46	48.36	44.22	53.24
150	28.31	26.20	22.71	22.79	31.98	32.07	32.53	29.83	54.50	45.50	48.96	39.39	48.14	44.17	53.30
151	28.14	26.27	22.84	22.74	31.34	32.41	33.02	29.69	54.40	45.60	49.00	39.40	48.36	44.20	53.39
152	28.74	26.18	22.42	22.65	33.22	32.17	31.26	29.65	54.90	45.10	48.65	39.49	47.09	44.07	53.19
153	28.39	26.18	22.80	22.63	32.29	31.93	32.51	29.58	54.60	45.40	48.87	39.42	48.00	44.14	53.88
154	28.46	26.27	22.72	22.54	32.55	32.20	32.26	29.28	54.70	45.30	48.87	39.36	47.57	44.12	53.83
155	28.40	26.02	22.73	22.84	32.23	31.35	32.31	30.53	54.40	45.60	48.88	39.22	48.62	44.05	54.43
156	28.44	26.15	22.66	22.74	32.69	31.99	31.38	29.84	54.60	45.40	48.80	39.90	47.52	44.35	53.36
157	28.46	26.25	22.61	22.68	32.79	32.27	31.12	29.68	54.70	45.30	48.74	39.92	47.20	44.33	53.34
158	28.52	26.24	22.57	22.67	33.02	32.19	30.96	29.67	54.80	45.20	48.71	39.92	47.09	44.32	53.20
159	28.54	26.25	22.55	22.66	33.04	32.11	30.98	29.80	54.80	45.20	48.62	39.84	47.17	44.23	53.36
160	28.22	26.16	22.74	22.87	31.72	32.11	31.69	30.50	54.40	45.60	48.96	39.65	48.23	44.30	53.77
161	28.23	26.25	22.71	22.81	31.79	32.37	31.47	30.32	54.50	45.50	48.96	39.65	47.94	44.30	53.70
162	28.31	25.77	22.68	23.24	32.78	30.50	30.95	31.62	54.10	45.90	49.09	39.90	48.77	44.49	53.51
163	28.04	26.41	22.91	22.64	31.50	32.82	31.90	29.55	54.40	45.60	49.04	39.90	47.71	44.47	53.93
164	28.21	26.56	22.61	22.61	32.10	33.51	30.93	29.16	54.80	45.20	49.03	40.00	46.66	44.52	53.66
165	27.97	26.40	23.00	22.63	31.18	32.83	32.24	29.53	54.40	45.60	49.07	39.87	47.94	44.47	53.85
166	28.33	26.56	22.62	22.49	32.49	33.13	30.97	29.15	54.90	45.10	48.77	39.88	46.69	44.33	53.87
167	28.43	25.70	22.66	23.20	33.57	30.30	30.79	31.18	54.10	45.90	49.39	39.89	48.32	44.64	53.96
168	28.16	25.97	22.86	23.01	32.43	31.52	31.60	30.20	54.10	45.90	49.46	40.05	48.10	44.76	54.08
169	28.31	26.43	22.65	22.59	32.06	32.91	31.82	29.40	54.80	45.20	48.75	39.62	47.33	44.19	53.49
170	28.35	26.42	22.61	22.60	32.19	32.88	31.71	29.44	54.80	45.20	48.75	39.59	47.28	44.17	53.48
171	28.43	25.80	22.60	23.17	33.12	30.69	30.92	31.24	54.20	45.80	49.15	39.79	48.39	44.47	54.03
172	28.44	25.80	22.60	23.16	33.16	30.69	30.92	31.20	54.20	45.80	49.15	39.79	48.36	44.47	54.03

173	28.27	25.85	22.75	23.13	32.60	30.97	31.60	30.83	54.10	45.90	49.26	39.84	48.54	44.55	54.06
174	28.30	25.85	22.70	23.15	32.62	30.82	31.47	31.12	54.20	45.80	49.13	39.75	48.67	44.44	54.05
175	28.32	25.87	22.70	23.11	32.69	30.89	31.47	30.99	54.20	45.80	49.13	39.75	48.55	44.44	54.15
176	28.25	25.87	22.76	23.12	32.54	31.09	31.67	30.72	54.10	45.90	49.26	39.90	48.48	44.58	54.15
177	28.39	25.75	22.64	23.22	33.01	30.53	31.11	31.35	54.10	45.90	49.17	39.79	48.62	44.48	54.20
178	28.38	25.75	22.65	23.22	32.97	30.52	31.15	31.34	54.10	45.90	49.15	39.81	48.65	44.48	54.19
179	28.34	25.78	22.68	23.20	32.82	30.63	31.29	31.27	54.10	45.90	49.20	39.76	48.68	44.48	54.19
180	28.35	25.82	22.67	23.16	32.84	30.75	31.29	31.12	54.20	45.80	49.19	39.75	48.55	44.47	54.16
181	28.34	25.79	22.68	23.19	32.82	30.66	31.29	31.24	54.10	45.90	49.20	39.76	48.65	44.48	54.18
182	28.39	25.76	22.64	23.21	32.97	30.57	31.15	31.32	54.10	45.90	49.17	39.76	48.62	44.47	54.15
183	28.26	25.87	22.76	23.11	32.53	31.03	31.70	30.76	54.10	45.90	49.23	39.84	48.54	44.54	54.12
184	28.47	25.85	22.59	23.09	33.25	31.01	31.03	30.70	54.30	45.70	49.20	39.81	48.02	44.51	54.16
185	28.34	25.81	22.68	23.17	32.82	30.73	31.29	31.17	54.10	45.90	49.20	39.76	48.59	44.48	54.17
186	28.27	25.85	22.75	23.13	32.60	31.00	31.60	30.80	54.10	45.90	49.29	39.84	48.51	44.57	54.03
187	28.39	25.60	22.72	23.30	32.89	30.20	31.47	31.46	54.00	46.00	49.26	39.81	48.97	44.54	54.20
188	28.43	25.62	22.67	23.29	32.93	30.34	31.29	31.43	54.00	46.00	49.20	39.84	48.83	44.52	54.19
189	28.38	25.79	22.64	23.19	32.97	30.63	31.12	31.27	54.20	45.80	49.15	39.79	48.57	44.47	54.14
190	28.36	25.86	22.70	23.09	32.77	31.05	31.54	30.71	54.20	45.80	49.26	39.73	48.36	44.49	54.40
191	28.27	25.83	22.75	23.15	32.53	30.83	31.52	31.10	54.10	45.90	49.12	39.87	48.71	44.49	54.34
192	28.27	25.74	22.73	23.26	32.47	30.62	31.63	31.34	54.00	46.00	49.23	39.76	48.97	44.49	54.47
193	28.27	25.84	22.76	23.13	32.49	30.97	31.59	30.93	54.10	45.90	49.12	39.93	48.62	44.52	54.32
194	28.24	25.88	22.77	23.11	32.45	31.07	31.74	30.76	54.10	45.90	49.23	39.84	48.57	44.54	54.10
195	28.34	25.87	22.71	23.09	32.69	31.05	31.58	30.75	54.20	45.80	49.23	39.73	48.42	44.48	54.38
196	28.40	25.83	22.61	23.17	33.02	30.74	31.00	31.21	54.20	45.80	49.13	39.77	48.43	44.45	54.25
197	28.47	25.70	22.65	23.18	33.51	30.72	30.93	30.85	54.20	45.80	49.57	39.83	48.09	44.70	53.92

198	28.45	25.71	22.66	23.18	33.43	30.75	30.97	30.85	54.20	45.80	49.57	39.83	48.12	44.70	53.91
199	28.32	25.77	22.64	23.26	32.69	30.68	31.23	31.36	54.10	45.90	49.22	39.77	48.73	44.50	54.53
200	28.23	25.83	22.80	23.13	32.40	30.95	31.70	30.89	54.10	45.90	49.20	39.93	48.68	44.57	54.30
201	28.46	25.72	22.60	23.21	33.43	30.85	30.89	30.85	54.20	45.80	49.57	39.80	48.06	44.68	53.97
202	28.45	25.85	22.57	23.12	33.06	30.99	31.08	30.82	54.30	45.70	49.10	39.84	48.15	44.47	53.80
203	28.27	25.71	22.74	23.27	32.49	30.53	31.66	31.35	54.00	46.00	49.22	39.80	49.02	44.51	54.51
204	28.27	25.85	22.77	23.11	32.53	30.97	31.59	30.90	54.10	45.90	49.15	39.90	48.59	44.52	54.32
205	28.32	25.79	22.68	23.21	32.74	30.66	31.29	31.31	54.10	45.90	49.20	39.76	48.71	44.48	54.20
206	28.30	25.85	22.70	23.15	32.62	30.82	31.47	31.12	54.20	45.80	49.13	39.75	48.67	44.44	54.07
207	28.31	25.86	22.72	23.11	32.64	30.86	31.54	31.00	54.20	45.80	49.13	39.75	48.61	44.44	54.14
208	28.34	25.81	22.69	23.16	32.65	30.87	31.47	31.04	54.10	45.90	49.12	39.84	48.59	44.48	54.39
209	28.43	25.56	22.68	23.34	32.98	30.14	31.40	31.53	54.00	46.00	49.26	39.81	48.97	44.54	54.25
210	28.36	25.57	22.75	23.33	32.78	30.20	31.57	31.46	53.90	46.10	49.29	39.87	49.06	44.58	54.25
211	28.40	25.57	22.70	23.34	32.98	30.08	31.39	31.54	54.00	46.00	49.23	39.87	49.00	44.55	54.25
212	28.36	25.83	22.68	23.13	32.84	30.89	31.30	30.95	54.20	45.80	49.15	39.87	48.42	44.51	54.30
213	28.22	25.84	22.82	23.11	32.34	30.93	31.84	30.90	54.10	45.90	49.15	39.90	48.77	44.52	54.29
214	28.21	25.85	22.80	23.13	32.34	30.90	31.86	30.93	54.10	45.90	49.17	39.84	48.80	44.51	54.12
215	28.36	25.78	22.67	23.19	32.82	30.63	31.33	31.24	54.10	45.90	49.15	39.76	48.68	44.45	54.20
216	28.31	25.86	22.71	23.11	32.65	30.87	31.51	31.01	54.20	45.80	49.13	39.75	48.58	44.44	54.14
217	28.38	25.76	22.68	23.18	33.01	30.52	31.25	31.24	54.10	45.90	49.17	39.79	48.62	44.48	54.17
218	28.21	25.84	22.80	23.15	32.38	30.86	31.82	30.97	54.10	45.90	49.20	39.84	48.80	44.52	54.19
219	28.21	25.84	22.81	23.13	32.34	30.86	31.91	30.93	54.10	45.90	49.17	39.84	48.83	44.51	54.13
220	28.40	25.76	22.65	23.19	33.04	30.52	31.17	31.27	54.20	45.80	49.15	39.79	48.59	44.47	54.16
221	28.38	25.78	22.67	23.17	32.97	30.56	31.25	31.24	54.20	45.80	49.12	39.79	48.62	44.45	54.21
222	28.39	25.78	22.67	23.16	33.01	30.59	31.25	31.17	54.20	45.80	49.15	39.79	48.57	44.47	54.15

223	28.33	25.82	22.70	23.15	32.78	30.76	31.37	31.10	54.10	45.90	49.20	39.76	48.59	44.48	54.12
224	28.20	25.82	22.80	23.18	32.37	30.80	31.81	31.03	54.00	46.00	49.20	39.87	48.86	44.54	54.20
225	28.30	25.87	22.73	23.10	32.64	31.14	31.59	30.66	54.20	45.80	49.26	39.87	48.36	44.57	54.13
226	28.35	25.78	22.67	23.20	32.82	30.63	31.29	31.27	54.10	45.90	49.17	39.76	48.68	44.47	54.14
227	28.38	25.78	22.63	23.21	32.93	30.67	31.11	31.28	54.20	45.80	49.17	39.79	48.57	44.48	54.11
228	28.21	25.85	22.80	23.14	32.42	30.90	31.78	30.93	54.10	45.90	49.23	39.84	48.74	44.54	54.18
229	28.42	25.82	22.65	23.11	33.15	30.93	31.13	30.73	54.20	45.80	49.29	39.87	48.13	44.58	54.33
230	28.39	25.75	22.63	23.22	32.98	30.58	31.12	31.33	54.10	45.90	49.17	39.79	48.59	44.48	54.17
231	28.51	25.91	22.58	23.00	33.69	30.90	30.55	30.70	54.40	45.60	49.10	39.92	47.71	44.51	53.34
232	28.37	25.83	22.75	23.05	33.11	30.92	31.36	30.52	54.20	45.80	49.19	40.06	48.15	44.63	53.68
233	28.45	25.66	22.62	23.27	33.42	30.48	30.91	31.16	54.10	45.90	49.49	39.85	48.33	44.67	54.09
234	28.37	25.65	22.66	23.32	33.17	30.31	31.05	31.40	54.00	46.00	49.38	39.90	48.65	44.64	54.08
235	28.53	25.63	22.58	23.26	33.66	30.27	30.73	31.25	54.20	45.80	49.26	39.97	48.31	44.61	53.93
236	28.39	25.71	22.68	23.22	33.23	30.67	31.06	30.91	54.10	45.90	49.38	40.05	48.28	44.71	53.78
237	28.47	25.69	22.59	23.26	33.43	30.44	30.85	31.22	54.20	45.80	49.32	39.88	48.33	44.60	54.14
238	28.21	26.21	22.70	22.87	32.63	32.27	30.90	29.93	54.40	45.60	49.35	40.02	47.35	44.68	53.86
239	28.19	26.20	22.69	22.91	32.63	32.28	30.90	29.94	54.40	45.60	49.43	40.02	47.35	44.73	53.87
240	28.44	26.03	22.63	22.89	32.85	31.73	30.94	30.26	54.50	45.50	49.23	39.74	47.61	44.49	53.57
241	28.46	26.00	22.63	22.91	32.81	31.67	31.02	30.34	54.50	45.50	49.22	39.67	47.71	44.44	53.51
242	28.56	26.10	22.51	22.83	33.15	31.93	30.66	30.19	54.70	45.30	49.22	39.55	47.28	44.38	53.56
243	28.61	26.13	22.47	22.80	33.25	31.98	30.58	30.11	54.70	45.30	49.15	39.51	47.15	44.33	53.60
244	28.49	26.04	22.60	22.87	33.01	31.67	30.91	30.24	54.50	45.50	49.15	39.71	47.55	44.43	53.60
245	28.38	26.13	22.61	22.87	32.71	32.03	30.95	30.16	54.50	45.50	49.25	39.72	47.48	44.48	53.65
246	28.46	26.19	22.58	22.77	33.06	32.32	30.72	29.63	54.70	45.30	49.31	39.81	46.93	44.56	53.40
247	28.41	26.21	22.61	22.77	32.87	32.32	30.87	29.67	54.60	45.40	49.25	39.81	47.08	44.53	53.61

248	28.59	25.89	22.47	23.05	33.40	31.12	30.37	30.95	54.50	45.50	49.15	39.65	47.76	44.40	53.39
249	28.41	26.07	22.65	22.84	32.77	31.73	31.04	30.33	54.50	45.50	49.12	39.65	47.70	44.38	53.88
250	28.47	26.24	22.58	22.71	32.95	32.37	30.84	29.62	54.70	45.30	49.19	39.69	46.99	44.44	53.63
251	28.63	26.15	22.48	22.74	33.40	32.18	30.59	29.76	54.80	45.20	49.25	39.52	46.87	44.38	53.47
252	28.46	26.21	22.57	22.76	33.13	32.35	30.57	29.57	54.70	45.30	49.33	39.84	46.82	44.59	53.63
253	28.48	26.24	22.56	22.72	33.10	32.46	30.69	29.47	54.70	45.30	49.31	39.75	46.79	44.53	53.58
254	28.48	26.21	22.57	22.74	33.13	32.36	30.69	29.54	54.70	45.30	49.33	39.75	46.84	44.54	53.63
255	28.47	26.20	22.56	22.77	33.15	32.35	30.59	29.57	54.70	45.30	49.33	39.84	46.82	44.59	53.63
256	28.43	26.22	22.62	22.73	32.93	32.35	30.98	29.53	54.70	45.30	49.31	39.72	47.02	44.51	53.60
257	28.44	26.22	22.59	22.74	32.97	32.44	30.80	29.52	54.70	45.30	49.29	39.80	46.89	44.54	53.65
258	28.41	26.33	22.60	22.66	32.82	32.62	30.98	29.36	54.70	45.30	49.13	39.78	46.87	44.46	53.63
259	28.49	26.26	22.54	22.71	33.13	32.53	30.62	29.44	54.70	45.30	49.28	39.78	46.70	44.53	53.56
260	28.19	26.53	22.64	22.64	31.99	33.54	31.05	29.15	54.70	45.30	49.13	39.98	46.73	44.56	53.81
261	28.50	26.19	22.53	22.78	33.16	32.25	30.55	29.77	54.70	45.30	49.23	39.77	46.92	44.50	53.34
262	28.49	26.20	22.56	22.75	33.07	32.24	30.72	29.73	54.70	45.30	49.22	39.73	46.99	44.47	53.35
263	28.49	26.21	22.54	22.76	33.12	32.28	30.64	29.70	54.70	45.30	49.20	39.77	46.92	44.49	53.40
264	28.47	26.19	22.57	22.77	33.11	32.28	30.61	29.70	54.70	45.30	49.29	39.80	46.92	44.54	53.48
265	28.45	26.23	22.56	22.76	33.05	32.26	30.58	29.82	54.70	45.30	49.19	39.78	46.99	44.48	53.50
266	28.45	26.21	22.57	22.77	33.08	32.32	30.62	29.67	54.70	45.30	49.31	39.81	46.90	44.56	53.47
267	28.17	26.54	22.68	22.60	31.86	33.47	31.23	29.18	54.70	45.30	49.00	39.97	46.89	44.49	53.66
268	28.35	25.81	22.66	23.18	33.08	30.77	31.04	30.97	54.20	45.80	49.29	39.97	48.28	44.63	54.32
269	28.56	26.03	22.56	22.85	33.85	31.30	30.50	30.24	54.60	45.40	49.19	39.78	47.28	44.48	53.44
270	28.38	25.72	22.71	23.19	33.12	30.55	31.28	31.06	54.10	45.90	49.32	39.87	48.51	44.60	54.25
271	28.27	25.98	22.69	23.06	32.84	31.57	30.91	30.45	54.30	45.70	49.41	40.05	47.78	44.73	53.35
272	28.47	26.16	22.55	22.82	33.02	32.13	30.72	29.86	54.60	45.40	49.18	39.80	47.12	44.49	53.59

273	28.50	26.06	22.55	22.89	33.15	31.66	30.63	30.40	54.60	45.40	49.20	39.65	47.47	44.43	53.62
274	28.57	25.61	22.71	23.11	33.60	30.51	31.17	30.65	54.20	45.80	49.58	39.78	48.10	44.68	54.67
275	28.06	26.10	22.83	23.01	31.46	31.43	31.91	31.09	54.20	45.80	48.67	39.87	48.98	44.27	54.32
276	28.14	26.42	22.93	22.51	31.92	32.79	31.98	29.12	54.60	45.40	48.96	39.95	47.39	44.46	53.94
277	28.61	25.93	22.50	22.96	33.97	30.97	30.30	30.63	54.50	45.50	49.15	39.76	47.47	44.45	53.42
278	28.61	25.94	22.50	22.95	33.99	30.98	30.26	30.64	54.50	45.50	49.13	39.77	47.45	44.45	53.50
279	28.39	26.03	22.67	22.92	33.01	31.31	31.39	30.36	54.40	45.60	49.15	39.68	47.93	44.41	53.96
280	28.14	26.06	22.77	23.02	32.20	31.77	31.49	30.41	54.20	45.80	49.38	39.88	48.13	44.63	54.05
281	28.20	26.13	22.73	22.94	32.43	31.89	31.33	30.23	54.30	45.70	49.33	39.83	47.86	44.58	53.95
282	28.13	26.10	22.81	22.96	32.28	31.94	31.45	30.14	54.20	45.80	49.49	39.91	47.90	44.70	53.98
283	28.09	26.11	22.84	22.96	32.22	31.90	31.57	30.13	54.20	45.80	49.46	39.94	47.99	44.70	54.06
284	28.14	26.11	22.77	22.98	32.22	31.84	31.51	30.34	54.30	45.70	49.35	39.82	48.07	44.58	53.97
285	28.14	26.06	22.75	23.05	32.34	31.67	31.36	30.41	54.20	45.80	49.33	39.98	48.09	44.66	54.09
286	28.17	26.02	22.78	23.03	32.37	31.45	31.43	30.60	54.20	45.80	49.25	39.92	48.26	44.58	53.91
287	28.14	26.03	22.80	23.03	32.29	31.45	31.50	30.60	54.20	45.80	49.25	39.92	48.32	44.58	53.89
288	28.22	26.08	22.74	22.96	32.48	31.59	31.37	30.43	54.30	45.70	49.13	39.92	48.06	44.52	53.85
289	28.09	26.01	22.83	23.07	32.14	31.44	31.54	30.70	54.10	45.90	49.25	40.01	48.44	44.63	54.01
290	28.10	25.99	22.82	23.09	32.18	31.38	31.50	30.76	54.10	45.90	49.25	40.01	48.46	44.63	54.01
291	28.08	26.02	22.82	23.08	32.22	31.48	31.49	30.63	54.10	45.90	49.33	40.01	48.35	44.67	54.03
292	28.07	26.04	22.83	23.06	31.99	31.60	31.67	30.59	54.10	45.90	49.36	39.87	48.44	44.62	54.11
293	28.06	26.07	22.85	23.02	31.95	31.64	31.75	30.52	54.10	45.90	49.31	39.87	48.44	44.59	54.14
294	28.21	26.01	22.75	23.04	32.57	31.43	31.23	30.62	54.20	45.80	49.33	39.87	48.15	44.60	54.05
295	28.23	25.99	22.69	23.09	32.52	31.42	31.12	30.78	54.20	45.80	49.25	39.90	48.20	44.57	54.05
296	28.17	25.95	22.80	23.07	32.53	31.34	31.31	30.63	54.10	45.90	49.48	39.92	48.23	44.70	54.20
297	28.17	25.96	22.80	23.07	32.53	31.38	31.31	30.59	54.10	45.90	49.48	39.92	48.20	44.70	54.16

298	28.16	25.93	22.81	23.09	32.53	31.29	31.32	30.64	54.10	45.90	49.48	39.98	48.26	44.73	54.11
299	28.20	25.93	22.79	23.08	32.54	31.32	31.36	30.61	54.10	45.90	49.45	39.92	48.23	44.69	54.22
300	28.19	25.93	22.79	23.09	32.57	31.33	31.31	30.62	54.10	45.90	49.51	39.92	48.20	44.72	54.38
301	28.18	25.93	22.79	23.09	32.48	31.36	31.39	30.62	54.10	45.90	49.51	39.90	48.26	44.70	54.19
302	28.20	26.05	22.75	23.00	32.55	31.61	31.22	30.52	54.20	45.80	49.36	39.86	48.03	44.61	53.96
303	28.24	26.16	22.74	22.86	32.55	32.01	31.30	30.04	54.40	45.60	49.25	39.89	47.65	44.57	54.01
304	28.18	26.19	22.85	22.78	32.37	32.10	31.54	29.86	54.40	45.60	49.23	39.97	47.70	44.60	53.90
305	28.27	26.12	22.76	22.84	32.67	31.91	31.33	30.04	54.40	45.60	49.29	39.85	47.67	44.57	54.00
306	28.26	26.12	22.75	22.87	32.63	31.96	31.26	30.06	54.40	45.60	49.32	39.91	47.64	44.61	54.00
307	28.24	26.14	22.80	22.82	32.43	32.07	31.49	29.93	54.40	45.60	49.29	39.88	47.70	44.58	54.05
308	28.46	26.15	22.58	22.82	33.22	31.99	30.58	30.05	54.60	45.40	49.04	39.98	47.16	44.51	53.81
309	28.46	26.05	22.61	22.88	33.17	31.65	30.76	30.29	54.50	45.50	49.00	39.97	47.49	44.48	53.82
310	28.18	26.12	22.81	22.89	32.28	31.87	31.42	30.24	54.30	45.70	49.09	40.05	47.96	44.57	53.81
311	28.29	26.20	22.78	22.74	32.51	32.08	31.43	29.90	54.50	45.50	48.96	39.95	47.63	44.46	53.69
312	28.34	26.20	22.68	22.78	33.02	31.91	31.18	29.78	54.50	45.50	49.04	39.95	47.37	44.50	53.71
313	28.33	26.21	22.69	22.77	33.01	31.98	31.15	29.71	54.50	45.50	49.10	39.95	47.31	44.53	53.70
314	28.31	26.27	22.68	22.75	32.89	32.22	31.11	29.61	54.60	45.40	49.13	39.95	47.19	44.54	53.88
315	28.34	26.27	22.63	22.76	33.01	32.22	30.91	29.67	54.60	45.40	49.10	39.95	47.10	44.53	53.64
316	28.35	26.25	22.62	22.78	33.07	32.06	30.94	29.76	54.60	45.40	49.04	39.95	47.19	44.50	53.68
317	28.44	26.28	22.60	22.67	33.12	32.16	31.14	29.58	54.70	45.30	49.00	39.71	47.12	44.36	53.81
318	28.43	26.19	22.59	22.79	33.17	31.91	31.01	29.84	54.60	45.40	49.04	39.81	47.28	44.43	53.70
319	28.36	26.20	22.66	22.77	32.89	31.97	31.36	29.76	54.60	45.40	49.06	39.80	47.44	44.43	53.90
320	28.32	26.19	22.72	22.77	32.78	31.92	31.47	29.79	54.50	45.50	49.12	39.80	47.55	44.46	53.84
321	28.40	26.23	22.60	22.77	33.28	31.97	30.72	29.86	54.60	45.40	49.12	39.88	47.12	44.50	53.76
322	28.41	26.24	22.60	22.75	33.32	31.99	30.72	29.79	54.60	45.40	49.09	39.91	47.06	44.50	53.76

323	28.36	26.21	22.63	22.80	33.15	32.01	30.77	29.87	54.60	45.40	49.17	39.94	47.18	44.56	53.82
324	28.41	26.18	22.59	22.82	33.23	31.82	30.75	30.05	54.60	45.40	49.15	39.79	47.29	44.47	53.76
325	28.25	26.28	22.65	22.81	32.66	32.62	30.94	29.60	54.50	45.50	49.41	39.93	47.06	44.67	53.58
326	28.44	26.03	22.49	23.04	33.36	31.61	30.29	30.56	54.50	45.50	49.30	39.89	47.39	44.60	53.96
327	28.26	26.04	22.76	22.94	32.71	31.70	31.11	30.28	54.30	45.70	49.17	40.13	47.78	44.65	53.90
328	28.54	26.22	22.36	22.89	33.66	32.16	29.74	30.12	54.80	45.20	49.06	40.02	46.66	44.54	53.78
329	28.54	26.21	22.34	22.90	33.63	32.18	29.75	30.14	54.80	45.20	49.07	40.01	46.67	44.54	53.81
330	28.54	26.23	22.34	22.88	33.69	32.25	29.68	30.07	54.80	45.20	49.09	40.02	46.57	44.55	53.69
331	28.56	26.20	22.33	22.91	33.71	32.17	29.67	30.13	54.80	45.20	49.07	40.03	46.61	44.55	53.78
332	28.54	26.19	22.33	22.93	33.66	32.17	29.74	30.17	54.70	45.30	49.16	39.98	46.67	44.57	53.86
333	28.47	26.23	22.41	22.89	33.37	32.20	30.04	30.10	54.70	45.30	49.07	40.01	46.84	44.54	53.80
334	28.47	26.05	22.59	22.89	33.11	31.59	30.87	30.33	54.50	45.50	48.97	39.85	47.61	44.41	53.48
335	28.45	26.04	22.61	22.89	33.06	31.51	30.97	30.39	54.50	45.50	48.97	39.83	47.73	44.40	53.52
336	28.45	26.03	22.60	22.91	33.06	31.48	30.92	30.46	54.50	45.50	48.97	39.83	47.76	44.40	53.60
337	28.45	26.08	22.60	22.88	33.06	31.66	30.84	30.37	54.50	45.50	49.00	39.80	47.61	44.40	53.43
338	28.34	26.15	22.66	22.84	32.76	31.86	31.09	30.20	54.50	45.50	49.07	39.78	47.65	44.43	53.48
339	28.48	26.05	22.53	22.93	33.32	31.50	30.59	30.51	54.50	45.50	49.00	39.84	47.55	44.42	53.33
340	28.46	26.05	22.56	22.93	33.21	31.50	30.71	30.51	54.50	45.50	49.00	39.84	47.64	44.42	53.41
341	28.45	26.06	22.56	22.93	33.17	31.53	30.71	30.51	54.50	45.50	49.00	39.84	47.64	44.42	53.36
342	28.30	25.99	22.65	23.06	32.73	31.41	31.00	30.76	54.30	45.70	49.17	39.87	48.07	44.52	53.52
343	28.47	26.01	22.57	22.94	33.17	31.39	30.82	30.50	54.50	45.50	48.97	39.84	47.73	44.41	53.75
344	28.47	26.02	22.59	22.92	33.13	31.43	30.91	30.41	54.50	45.50	48.96	39.86	47.71	44.41	53.81
345	28.51	25.95	22.57	22.98	33.20	31.23	30.89	30.62	54.50	45.50	48.99	39.80	47.86	44.39	53.86
346	28.49	25.98	22.59	22.94	33.20	31.38	30.89	30.42	54.50	45.50	49.02	39.86	47.71	44.44	53.80
347	28.48	25.89	22.61	23.03	33.25	30.99	30.90	30.75	54.40	45.60	49.04	39.86	48.00	44.45	53.51

348	28.45	25.93	22.63	22.99	33.21	31.07	30.95	30.63	54.40	45.60	49.04	39.89	47.94	44.47	53.51
349	28.37	25.90	22.73	23.00	32.93	31.00	31.27	30.66	54.30	45.70	49.07	39.92	48.20	44.50	53.68
350	28.33	25.95	22.70	23.02	32.79	31.23	31.14	30.65	54.30	45.70	49.16	39.89	48.12	44.52	53.66
351	28.30	26.13	22.66	22.90	32.79	31.59	30.96	30.47	54.40	45.60	48.96	39.92	47.83	44.44	53.54
352	28.43	26.00	22.57	23.00	33.16	31.21	30.82	30.66	54.40	45.60	48.96	39.86	47.89	44.41	53.38
353	28.40	26.06	22.52	23.02	33.26	31.60	30.43	30.48	54.50	45.50	49.26	39.90	47.46	44.58	53.79
354	28.37	26.00	22.57	23.06	33.23	31.46	30.51	30.57	54.40	45.60	49.38	39.90	47.61	44.64	53.83
355	28.31	25.98	22.61	23.09	33.04	31.42	30.60	30.64	54.30	45.70	49.35	40.02	47.75	44.68	53.93
356	28.35	25.95	22.58	23.11	33.13	31.43	30.53	30.65	54.30	45.70	49.41	39.99	47.70	44.70	53.95
357	28.27	25.94	22.71	23.08	32.68	31.33	31.15	30.75	54.20	45.80	49.42	39.77	48.17	44.60	53.59
358	28.30	25.95	22.66	23.09	32.86	31.38	30.87	30.73	54.20	45.80	49.39	39.89	47.97	44.64	53.66
359	28.51	25.87	22.46	23.16	33.84	30.68	30.12	31.18	54.40	45.60	49.36	39.69	47.80	44.53	54.20
360	28.50	25.88	22.46	23.16	33.80	30.71	30.11	31.18	54.40	45.60	49.33	39.72	47.80	44.53	54.19
361	28.34	25.92	22.66	23.08	33.02	31.03	31.06	30.83	54.30	45.70	49.25	39.83	48.15	44.54	54.17
362	28.43	26.05	22.59	22.94	33.28	31.53	30.57	30.48	54.50	45.50	49.25	39.83	47.51	44.54	53.72
363	28.40	25.67	22.73	23.20	33.18	30.56	31.35	30.87	54.10	45.90	49.38	39.99	48.42	44.68	53.90
364	28.30	25.72	22.77	23.21	32.97	30.62	31.36	30.93	54.00	46.00	49.38	40.05	48.51	44.71	54.03
365	28.30	25.87	22.70	23.13	32.74	30.91	31.35	30.94	54.20	45.80	49.15	39.87	48.48	44.51	54.05
366	28.40	25.79	22.63	23.19	33.01	30.83	31.12	31.00	54.20	45.80	49.28	39.83	48.35	44.55	54.07
367	28.35	25.84	22.66	23.15	32.76	30.98	31.44	30.84	54.20	45.80	49.15	39.87	48.42	44.51	54.27
368	28.43	25.89	22.60	23.09	32.96	31.08	31.37	30.71	54.30	45.70	49.06	39.79	48.22	44.42	54.02
369	28.58	26.04	22.51	22.87	33.92	31.43	30.30	30.17	54.60	45.40	49.20	39.85	47.09	44.53	53.60
370	28.61	26.05	22.49	22.85	34.09	31.51	30.15	30.05	54.70	45.30	49.22	39.89	46.90	44.55	53.58
371	28.59	25.97	22.51	22.93	33.97	31.28	30.27	30.33	54.60	45.40	49.28	39.86	47.19	44.57	53.79
372	28.45	26.00	22.61	22.95	33.41	31.34	30.83	30.25	54.40	45.60	49.20	39.91	47.55	44.56	53.92

373	28.40	25.98	22.55	23.07	33.03	31.36	30.45	30.88	54.40	45.60	49.19	39.83	47.83	44.51	53.05
374	28.39	25.79	22.43	23.39	33.21	30.96	30.13	31.40	54.20	45.80	49.45	39.98	48.03	44.71	54.01
375	28.39	25.81	22.42	23.38	33.21	31.03	30.09	31.36	54.20	45.80	49.45	39.98	47.97	44.71	53.98
376	28.47	25.91	22.30	23.32	33.57	31.28	29.56	31.25	54.40	45.60	49.43	39.93	47.49	44.68	53.90
377	28.25	25.76	22.63	23.36	32.76	30.58	30.68	31.74	54.00	46.00	49.32	39.93	48.71	44.62	53.46
378	28.28	26.11	22.76	22.85	32.78	31.99	31.35	29.78	54.40	45.60	49.49	39.84	47.49	44.67	53.24
379	28.30	26.17	22.72	22.81	32.87	32.20	31.21	29.65	54.50	45.50	49.55	39.79	47.26	44.67	53.22
380	28.21	26.15	22.75	22.89	32.15	31.87	31.47	30.45	54.40	45.60	48.87	39.95	48.12	44.41	53.11
381	28.24	26.22	22.70	22.84	32.40	32.06	31.30	30.13	54.50	45.50	48.94	39.94	47.73	44.44	53.21
382	28.32	25.87	22.74	23.08	32.79	31.10	31.11	30.83	54.20	45.80	49.29	39.90	48.25	44.60	53.36
383	28.38	25.86	22.67	23.09	32.78	30.69	31.33	31.10	54.20	45.80	48.77	39.91	48.60	44.34	53.52
384	28.48	26.05	22.60	22.87	33.13	31.70	30.74	30.24	54.50	45.50	49.29	39.68	47.44	44.49	53.58
385	28.12	26.06	22.81	23.01	32.02	31.90	31.39	30.48	54.20	45.80	49.59	39.74	48.14	44.67	54.48
386	28.14	26.10	22.79	22.98	32.09	32.01	31.31	30.37	54.20	45.80	49.57	39.74	48.00	44.65	54.47
387	28.36	25.98	22.72	22.93	32.64	31.53	31.14	30.48	54.30	45.70	49.25	39.75	47.97	44.50	54.38
388	28.28	26.00	22.84	22.88	32.32	31.64	31.72	30.32	54.30	45.70	49.30	39.68	48.17	44.49	54.05
389	28.71	25.45	22.65	23.19	34.16	29.75	30.92	31.07	54.20	45.80	49.36	39.86	48.29	44.61	54.20
390	28.72	25.49	22.64	23.15	34.17	29.77	30.92	31.06	54.20	45.80	49.32	39.79	48.28	44.55	54.27
391	28.70	25.54	22.64	23.13	34.04	29.98	30.98	30.93	54.20	45.80	49.30	39.80	48.20	44.55	54.21
392	28.61	25.79	22.66	22.94	33.77	30.77	31.15	30.30	54.40	45.60	49.27	39.76	47.77	44.52	54.58
393	28.59	25.75	22.67	22.99	33.71	30.69	31.11	30.39	54.30	45.70	49.39	39.73	47.85	44.56	54.50
394	28.41	25.65	22.89	23.05	32.97	30.31	32.10	30.68	54.10	45.90	49.29	39.75	48.77	44.52	54.70
395	28.51	25.69	22.74	23.05	33.42	30.69	31.34	30.46	54.20	45.80	49.46	39.86	48.07	44.66	54.50
396	28.66	25.62	22.60	23.11	33.84	30.45	30.88	30.76	54.30	45.70	49.39	39.79	47.97	44.59	54.60
397	28.63	25.62	22.67	23.09	33.61	30.31	31.30	30.75	54.20	45.80	49.19	39.82	48.26	44.50	54.39

398	28.63	25.68	22.73	22.96	33.94	30.52	31.19	30.29	54.30	45.70	49.39	39.85	47.82	44.62	54.55
399	28.60	25.66	22.75	23.00	33.79	30.46	31.28	30.42	54.30	45.70	49.39	39.85	48.00	44.62	54.48
400	28.41	25.70	22.77	23.12	32.82	30.55	31.73	30.89	54.10	45.90	49.22	39.80	48.67	44.51	54.59
401	28.38	25.63	22.83	23.16	32.78	30.31	31.88	31.02	54.00	46.00	49.28	39.80	48.90	44.54	54.59
402	28.48	25.68	22.75	23.08	33.21	30.42	31.60	30.73	54.20	45.80	49.28	39.77	48.46	44.52	54.65
403	28.50	25.70	22.70	23.10	33.28	30.47	31.25	30.88	54.20	45.80	49.19	39.86	48.35	44.52	54.48
404	28.40	25.78	22.70	23.12	32.94	30.94	31.21	30.83	54.20	45.80	49.49	39.69	48.27	44.59	54.32
405	27.98	26.16	22.87	23.00	31.36	31.70	31.91	30.82	54.10	45.90	48.85	39.95	48.80	44.40	54.35
406	28.03	26.23	22.75	23.00	31.46	31.96	31.72	30.70	54.30	45.70	48.84	39.88	48.52	44.36	54.28
407	28.23	26.21	22.94	22.62	31.85	31.91	32.70	29.77	54.40	45.60	48.67	39.68	48.32	44.17	54.35
408	28.20	26.27	22.92	22.61	31.70	32.10	32.66	29.78	54.50	45.50	48.64	39.65	48.29	44.14	54.17
409	28.26	26.17	22.84	22.72	32.09	31.65	31.95	30.26	54.40	45.60	48.58	39.83	48.29	44.20	54.06
410	28.25	26.16	22.84	22.74	32.13	31.58	31.91	30.32	54.40	45.60	48.61	39.83	48.32	44.22	54.17
411	28.23	26.19	22.89	22.69	32.07	31.82	32.02	30.05	54.40	45.60	48.75	39.80	48.17	44.28	54.18
412	28.26	26.13	22.87	22.74	32.17	31.60	31.94	30.27	54.40	45.60	48.72	39.83	48.29	44.28	54.03
413	28.30	26.22	22.83	22.65	32.34	32.05	31.75	29.83	54.50	45.50	48.85	39.81	47.78	44.33	54.14
414	28.07	25.73	22.98	23.22	31.26	30.64	32.65	31.56	53.80	46.20	48.87	39.85	49.88	44.36	54.20
415	28.09	25.72	22.98	23.21	31.31	30.50	32.67	31.66	53.80	46.20	48.78	39.82	49.97	44.30	54.22
416	28.05	25.85	22.99	23.11	31.04	31.05	32.83	31.22	53.90	46.10	48.78	39.82	49.71	44.30	54.25
417	28.16	25.72	22.95	23.17	31.47	30.79	32.54	31.30	53.90	46.10	48.90	39.88	49.59	44.39	54.08
418	28.00	25.75	23.05	23.20	31.08	30.54	33.02	31.56	53.80	46.20	48.84	39.79	50.12	44.31	53.95
419	28.39	26.42	22.61	22.58	32.28	32.94	31.68	29.33	54.80	45.20	48.74	39.61	47.20	44.17	53.29
420	28.30	26.11	22.81	22.78	31.89	31.88	32.26	30.11	54.40	45.60	48.70	39.72	48.35	44.21	54.03
421	28.36	26.22	22.73	22.68	32.10	32.30	31.92	29.85	54.60	45.40	48.72	39.67	47.85	44.20	53.48
422	28.30	26.57	22.63	22.51	32.25	33.33	30.98	29.18	54.90	45.10	48.81	39.90	46.70	44.35	53.63

423	28.24	26.12	22.73	22.91	31.71	31.84	31.81	30.72	54.40	45.60	48.86	39.58	48.48	44.22	53.77
424	28.33	26.09	22.42	23.15	32.71	31.96	30.55	30.74	54.40	45.60	49.32	39.76	47.64	44.54	53.60
425	28.35	25.90	22.66	23.09	32.82	30.94	31.15	30.91	54.20	45.80	48.97	39.99	48.30	44.48	53.63
426	28.50	25.87	22.61	23.03	33.50	30.89	30.77	30.69	54.40	45.60	49.13	39.92	47.86	44.52	53.37
427	28.46	26.09	22.60	22.85	32.78	31.73	30.98	30.33	54.60	45.40	49.02	39.64	47.69	44.33	53.47
428	28.41	26.20	22.67	22.72	33.07	31.97	31.18	29.77	54.60	45.40	49.01	39.83	47.33	44.42	53.68
429	28.23	26.05	22.80	22.92	32.51	31.88	31.13	30.22	54.30	45.70	49.59	39.80	47.77	44.70	54.53
430	28.22	26.11	22.86	22.81	32.43	32.11	31.46	29.80	54.30	45.70	49.46	39.94	47.61	44.70	54.53
431	28.41	26.49	22.57	22.53	32.35	33.04	31.64	29.23	54.90	45.10	48.74	39.52	47.06	44.13	53.44
432	28.48	26.20	22.60	22.72	33.10	32.33	30.82	29.51	54.70	45.30	49.31	39.75	46.90	44.53	53.63
433	28.47	26.18	22.58	22.77	33.06	32.25	30.60	29.77	54.70	45.30	49.20	39.86	46.98	44.53	53.56
434	28.53	26.16	22.52	22.79	33.21	32.17	30.57	29.79	54.70	45.30	49.20	39.77	46.95	44.49	53.38
435	28.47	26.28	22.54	22.71	33.11	32.51	30.68	29.42	54.70	45.30	49.22	39.81	46.73	44.51	53.55
436	28.48	26.18	22.57	22.77	33.10	32.26	30.60	29.75	54.70	45.30	49.23	39.83	46.95	44.53	53.49
437	28.52	26.15	22.54	22.80	33.17	32.11	30.65	29.84	54.70	45.30	49.23	39.74	47.03	44.49	53.40
438	28.46	26.19	22.59	22.76	33.06	32.28	30.65	29.70	54.70	45.30	49.23	39.86	46.95	44.54	53.49
439	28.46	26.27	22.55	22.72	33.07	32.47	30.72	29.45	54.70	45.30	49.22	39.81	46.79	44.51	53.54
440	28.44	26.20	22.60	22.76	32.97	32.27	30.72	29.73	54.60	45.40	49.25	39.81	47.02	44.53	53.43
441	28.50	26.16	22.55	22.79	33.13	32.17	30.64	29.79	54.70	45.30	49.23	39.77	47.00	44.50	53.38
442	28.54	26.16	22.51	22.80	33.25	32.13	30.53	29.83	54.70	45.30	49.20	39.77	46.95	44.49	53.40
443	28.55	26.14	22.51	22.80	33.23	32.10	30.61	29.83	54.70	45.30	49.19	39.76	46.99	44.47	53.41
444	28.47	26.20	22.55	22.78	33.06	32.35	30.58	29.67	54.70	45.30	49.23	39.88	46.89	44.56	53.42
445	28.46	26.20	22.56	22.78	33.01	32.37	30.71	29.59	54.70	45.30	49.22	39.90	46.90	44.56	53.30
446	28.45	26.20	22.57	22.78	32.97	32.37	30.75	29.59	54.70	45.30	49.22	39.90	46.93	44.56	53.29
447	28.47	26.19	22.55	22.79	32.97	32.37	30.75	29.59	54.70	45.30	49.16	39.92	46.93	44.54	53.35

448	28.47	26.23	22.55	22.75	33.02	32.41	30.65	29.62	54.70	45.30	49.16	39.87	46.87	44.51	53.41
449	28.44	26.17	22.58	22.80	32.93	32.27	30.75	29.73	54.60	45.40	49.22	39.90	47.05	44.56	53.41
450	28.44	26.21	22.58	22.77	32.86	32.37	30.84	29.62	54.70	45.30	49.13	39.90	47.02	44.51	53.42
451	28.43	26.20	22.59	22.78	32.90	32.34	30.79	29.66	54.60	45.40	49.19	39.90	47.02	44.54	53.39
452	28.47	26.22	22.56	22.75	32.98	32.41	30.69	29.62	54.70	45.30	49.16	39.87	46.90	44.51	53.41
453	28.48	26.22	22.55	22.75	33.02	32.41	30.65	29.62	54.70	45.30	49.16	39.87	46.87	44.51	53.42
454	28.47	26.20	22.57	22.76	32.97	32.35	30.76	29.63	54.70	45.30	49.19	39.84	46.96	44.51	53.41
455	28.47	26.20	22.56	22.77	33.01	32.34	30.68	29.66	54.70	45.30	49.19	39.87	46.93	44.53	53.40
456	28.47	26.21	22.56	22.76	33.01	32.37	30.68	29.62	54.70	45.30	49.19	39.87	46.90	44.53	53.43
457	28.47	26.19	22.56	22.78	33.01	32.34	30.68	29.66	54.70	45.30	49.22	39.87	46.93	44.54	53.42
458	28.46	26.22	22.57	22.75	32.98	32.37	30.73	29.62	54.70	45.30	49.16	39.87	46.93	44.51	53.41
459	28.46	26.22	22.57	22.75	32.96	32.37	30.77	29.62	54.70	45.30	49.13	39.87	46.96	44.50	53.33
460	28.31	26.22	22.78	22.69	32.88	32.00	31.28	29.76	54.50	45.50	49.07	39.92	47.42	44.50	53.97
461	28.16	25.96	22.77	23.11	32.38	31.33	31.40	30.72	54.10	45.90	49.35	39.91	48.36	44.63	54.15
Avg	**28.34**	**26.03**	22.70	22.93	**32.69**	**31.58**	31.31	30.33	54.37	45.63	49.15	39.83	**47.91**	44.49	53.84
SD	0.15	0.23	0.16	0.20	0.63	0.76	0.68	0.62	0.27	0.27	0.22	0.16	0.71	0.15	0.35

ENC: effective number of codons; Avg: average; SD: standard deviation.

B. Nucleotide composition analysis of ABLV coding sequences (%).

No	A	U	G	C	A_3	U_3	G_3	C_3	AU	GC	GC_1	GC_2	GC_3	GC_{12}	ENC
1	29.00	27.16	21.84	22.00	33.01	35.22	30.50	27.95	56.20	43.80	47.77	38.90	44.85	43.33	53.15
2	29.08	26.51	21.66	22.75	33.17	32.91	30.42	30.18	55.60	44.40	47.54	39.07	46.62	43.31	54.26
3	29.05	27.11	21.82	22.01	33.12	35.19	30.51	27.91	56.20	43.80	47.76	38.94	44.81	43.35	53.13
4	29.11	27.00	21.76	22.13	33.37	34.71	30.26	28.36	56.10	43.90	47.70	38.96	45.01	43.33	53.16
5	29.13	26.98	21.76	22.13	33.45	34.57	30.21	28.46	56.10	43.90	47.64	38.96	45.07	43.30	53.16
6	29.07	26.96	21.81	22.16	33.22	34.71	30.42	28.36	56.00	44.00	47.82	38.96	45.13	43.39	53.19
Avg	**29.07**	**26.95**	21.78	22.20	**33.22**	**34.55**	30.39	28.54	56.03	43.97	47.71	38.97	45.25	43.34	53.34
SD	0.04	0.21	0.06	0.25	0.15	0.77	0.11	0.76	0.21	0.21	0.09	0.05	0.62	0.03	0.41

ENC: effective number of codons; Avg: average; SD: standard deviation.

C. Nucleotide composition analysis of DUVV coding sequences (%).

No	A	U	G	C	A_3	U_3	G_3	C_3	AU	GC	GC_1	GC_2	GC_3	GC_{12}	ENC
1	28.80	26.43	22.03	22.73	33.28	32.36	30.38	30.05	55.20	44.80	47.88	39.55	46.84	43.72	53.34
2	28.95	26.40	21.98	22.67	33.94	32.15	30.12	29.84	55.40	44.60	47.81	39.63	46.51	43.72	53.22
3	28.92	26.46	21.94	22.69	33.87	32.33	29.78	30.02	55.40	44.60	47.84	39.61	46.42	43.72	53.18
4	29.35	26.66	21.56	22.43	35.14	33.02	28.41	29.49	56.00	44.00	47.57	39.45	44.95	43.51	52.17
Avg	**29.01**	**26.49**	21.88	22.63	**34.06**	**32.47**	29.67	29.85	55.50	44.50	47.77	39.56	**46.18**	43.67	52.98
SD	0.21	0.10	0.18	0.12	0.68	0.33	0.76	0.22	0.30	0.30	0.12	0.07	0.73	0.09	0.47

ENC: effective number of codons; Avg: average; SD: standard deviation.

D. Nucleotide composition analysis of EBLV-1 coding sequences (%).

No	A	U	G	C	A_3	U_3	G_3	C_3	AU	GC	GC_1	GC_2	GC_3	GC_{12}	ENC
1	28.69	26.00	23.02	22.29	31.73	31.53	34.12	29.34	54.70	45.30	47.41	39.70	48.83	43.55	52.43
2	28.65	25.97	23.00	22.38	31.59	31.33	34.27	29.59	54.60	45.40	47.39	39.66	49.10	43.53	52.54
3	28.56	25.83	23.11	22.49	31.15	30.74	34.74	30.13	54.40	45.60	47.18	39.70	49.93	43.44	52.47
4	28.62	26.01	23.07	22.30	31.56	31.40	34.41	29.39	54.60	45.40	47.33	39.72	49.04	43.53	52.45
5	28.62	26.00	23.08	22.30	31.56	31.37	34.45	29.39	54.60	45.40	47.33	39.72	49.07	43.53	52.45
6	28.62	25.98	23.03	22.37	31.75	31.40	34.05	29.46	54.60	45.40	47.57	39.74	48.90	43.65	52.84
7	28.58	25.83	23.14	22.46	31.24	30.72	34.67	30.07	54.40	45.60	47.22	39.72	49.86	43.47	52.72
8	28.51	25.93	23.17	22.40	30.97	31.01	34.75	29.88	54.40	45.60	47.12	39.79	49.78	43.45	52.79
9	28.56	25.83	23.17	22.44	31.31	30.77	34.70	29.88	54.40	45.60	47.31	39.77	49.74	43.54	52.68
10	28.74	26.00	22.95	22.31	31.86	31.46	33.94	29.48	54.70	45.30	47.41	39.58	48.80	43.49	52.47
11	28.57	26.02	23.05	22.36	31.42	31.53	34.20	29.55	54.60	45.40	47.52	39.64	49.06	43.58	52.38
12	28.55	25.81	23.13	22.51	31.10	30.78	34.77	30.10	54.40	45.60	47.26	39.73	49.93	43.50	52.54
13	28.58	26.03	23.09	22.29	31.30	31.63	34.55	29.31	54.60	45.40	47.41	39.67	49.09	43.54	52.38
14	28.58	25.99	23.06	22.37	31.35	31.48	34.28	29.63	54.60	45.40	47.49	39.61	49.17	43.55	52.45
15	28.53	25.98	23.12	22.36	31.25	31.34	34.57	29.57	54.50	45.50	47.44	39.67	49.35	43.55	52.36
Avg	**28.60**	**25.95**	23.08	22.38	**31.41**	31.23	**34.43**	29.65	54.54	45.46	47.36	39.70	**49.31**	43.53	52.53
SD	0.06	0.08	0.06	0.07	0.25	0.32	0.27	0.28	0.11	0.11	0.12	0.06	0.40	0.05	0.15

ENC: effective number of codons; Avg: average; SD: standard deviation.

E. Nucleotide composition analysis of LBV coding sequences (%).

No	A	U	G	C	A_3	U_3	G_3	C_3	AU	GC	GC_1	GC_2	GC_3	GC_{12}	ENC
1	29.09	26.86	22.44	21.61	34.00	33.71	32.42	26.66	55.90	44.10	47.50	39.32	45.33	43.41	52.58
2	29.09	26.79	22.51	21.61	34.00	33.45	32.68	26.68	55.90	44.10	47.45	39.38	45.53	43.41	52.78
3	30.25	28.61	20.91	20.22	37.42	38.89	27.60	22.63	58.90	41.10	46.03	39.03	38.33	42.53	51.08
4	30.27	27.99	21.06	20.68	38.05	37.41	26.97	23.65	58.30	41.70	46.71	39.57	38.96	43.14	51.74
5	30.48	28.07	20.80	20.65	38.83	37.57	26.17	23.56	58.60	41.40	46.60	39.47	38.28	43.04	51.52
6	29.13	26.69	22.41	21.77	34.09	33.16	32.24	27.24	55.80	44.20	47.48	39.38	45.71	43.43	52.93
Avg	**29.72**	**27.50**	21.69	21.09	**36.07**	**35.70**	29.68	25.07	57.23	42.77	46.96	39.36	**42.02**	43.16	52.11
SD	0.62	0.75	0.77	0.60	2.08	2.31	2.80	1.83	1.38	1.38	0.55	0.17	3.51	0.32	0.69

ENC: effective number of codons; Avg: average; SD: standard deviation.

F. Nucleotide composition analysis of MOKV lyssavirus coding sequences (%).

No	A	U	G	C	A_3	U_3	G_3	C_3	AU	GC	GC_1	GC_2	GC_3	GC_{12}	ENC
1	28.45	26.06	22.78	22.71	32.33	31.56	32.23	30.03	54.50	45.50	48.58	39.66	48.23	44.12	53.76
2	28.67	26.72	22.40	22.21	33.35	33.12	30.84	28.82	55.40	44.60	47.95	39.76	46.12	43.86	53.00
3	28.84	26.35	22.52	22.29	33.35	32.91	31.24	28.79	55.20	44.80	48.62	39.44	46.36	44.03	52.69
4	28.82	26.38	22.50	22.30	33.26	33.02	31.22	28.76	55.20	44.80	48.62	39.44	46.33	44.03	52.64
5	28.71	26.50	22.54	22.25	32.94	33.07	31.44	28.95	55.20	44.80	48.78	38.99	46.61	43.88	52.67
6	28.98	26.18	22.21	22.62	34.43	31.26	30.04	30.41	55.20	44.80	48.10	39.49	46.91	43.80	52.74
Avg	**28.74**	**26.37**	22.49	22.40	**33.28**	**32.49**	31.17	29.29	55.12	44.88	48.44	39.46	**46.76**	43.95	52.92
SD	0.17	0.21	0.17	0.19	0.63	0.77	0.66	0.67	0.29	0.29	0.30	0.24	0.70	0.11	0.40

ENC: effective number of codons; Avg: average; SD: standard deviation.
