# Supplementary material for: Comprehensive Analysis of Codon Usage on Rabies Virus and Other Lyssaviruses
Source: Int J Mol Sci. 2018 Aug 14;19(8):2397. doi: 10.3390/ijms19082397 (PMC6121662; doi:10.3390/ijms19082397)
Supplement: Supplementary file 1 [file ijms-19-02397-s001.zip › Table S2 Characteristic of add lyssavirus individual gene coding sequences analysed in present study..pdf]

Supplemental table 2 Characteristic of add lyssavirus individual gene coding sequences analyzed in present study.

No	Accession	Host	Country	Year
1	U17064.1			
2	AF049113.1			
3	AF049114.1			
4	AF049115.1			
5	AF049116.1			
6	AF049117.1			
7	AF049120.1			
8	AF006497.1			
9	AF049119.2			
10	AF369369.1	Flying fox		

11	AF369370.1	Flying fox		
12	AF369371.1	Flying fox		
13	AF369372.1	Flying fox		
14	AF369373.1	insectivorous bat		
15	AF369374.1			
16	AF369375.1			
17	AF369376.1			
18	AF369377.1			
19	AF369378.1			
20	AF298146.1			
21	AF298147.1			
22	AF298148.1			

23	AF298149.1			
24	AF426290.1	Pteropus alecto		
25	AF426291.1	Pteropus poliocephalus		
26	AF426292.1	Pteropus scapulatus		
27	AF426293.1	Pteropus alecto		
28	AF426294.1	Pteropus alecto		
29	AF426295.1	Pteropus poliocephalus		
30	AF426296.1	Pteropus poliocephalus		
31	AF426297.1	Saccolaimus flaviventris		
32	AF426298.1	Saccolaimus flaviventris		
33	AF426299.1	Pteropus poliocephalus		
34	AF426300.1	Pteropus alecto		

35	AF426301.1	Saccolaimus flaviventris		
36	AF426302.1	Pteropus alecto		
37	AF426303.1	Pteropus scapulatus		
38	AF426304.1	Pteropus alecto		
39	AF426305.1	Pteropus scapulatus		
40	AF426306.1	Pteropus alecto		
41	AF426307.1	Pteropus alecto		
42	AF426308.1	Pteropus alecto		
43	AF426309.1	Pteropus alecto		
44	AF426310.1	Pteropus alecto		
45	AF426311.1	Pteropus alecto		
46	AF429312.1		Nigeria	1956

47	AY573935.1	Pteropus alecto		
48	AY573936.1	Pteropus alecto		
49	AY573937.1	Saccolaimus flaviventris		
50	AY573938.1	Pteropus alecto		
51	AY573939.1	Pteropus alecto		
52	AY573940.1	Pteropus alecto		
53	AY573941.1	Pteropus poliocephalus		
54	AY573942.1	Pteropus alecto		
55	AY573943.1	Pteropus alecto		
56	AY573944.1	Pteropus scapulatus		
57	AY573945.1	Pteropus poliocephalus		
58	AY573947.1	Pteropus alecto		

59	AY573948.1	Pteropus poliocephalus		
60	AY573949.1	Saccolaimus flaviventris		
61	AY573950.1	Pteropus poliocephalus		
62	AY573951.1	Pteropus scapulatus		
63	AY573952.1	Pteropus scapulatus		
64	AY573953.1	Pteropus scapulatus		
65	AY573954.1	Pteropus alecto		
66	AY573955.1	Pteropus scapulatus		
67	AY573957.1	Pteropus scapulatus		
68	AY573958.1	Pteropus poliocephalus		
69	AY573959.1	Pteropus alecto		
70	AY573960.1	Pteropus scapulatus		

71	AY573961.1	Pteropus alecto		
72	AY573962.1	Pteropus alecto		
73	AY573963.1	Pteropus alecto		
74	AY573965.1	Saccolaimus flaviventris		
75	DQ499944.1	Epomophorus whalbergi	South Africa	
76	DQ499945.1	Epomophorus whalbergi	South Africa	
77	DQ499946.1	Epomophorus whalbergi	South Africa	
78	DQ499947.1	Epomophorus whalbergi	South Africa	
79	DQ499948.1	Atilax paludinosus	South Africa	
80	EF547407.1		Nigeria	1956
81	EF547408.1		South Africa	1980
82	EF547409.1		South Africa	2004

83	EF547410.1		South Africa	1982
84	EF547411.1		South Africa	1980
85	EF547412.1		South Africa	1980
86	EF547413.1		South Africa	2003
87	EF547414.1		South Africa	2006
88	EF547415.1		South Africa	2004
89	EF547416.1		Zimbabwe	1986
90	EF547417.1		Central African Republic	1974
91	EF547418.1			1999
92	EF547419.1		Senegal	1985
93	EF547420.1		South Africa	2006

94	EF547434.1		South Africa	2003
95	EF547435.1		South Africa	2006
96	EF547436.1		South Africa	1980
97	EF547437.1		South Africa	1980
98	EF547438.1		South Africa	2004
99	EF547439.1		South Africa	1982
100	EF547440.1		South Africa	2004
101	EF547441.1		South Africa	1980
102	EF547442.1		Zimbabwe	1986
103	EF547444.1		Nigeria	1956
104	EF547445.1			1999
105	EF547446.1		Senegal	1985

106	EF547421.1		South Africa	2003
107	EF547422.1		South Africa	2006
108	EF547423.1		South Africa	2004
109	EF547424.1		South Africa	1980
110	EF547425.1		South Africa	1982
111	EF547426.1		South Africa	1980
112	EF547427.1		South Africa	1980
113	EF547428.1		South Africa	2004
114	EF547429.1		Zimbabwe	1986
115	EF547430.1		Central African Republic	1974
116	EF547431.1		Nigeria	1956

117	EF547432.1			1999
118	EF547433.1		Senegal	1985
119	EF547447.1	mouse		1999
120	EF547448.1	mouse	Senegal	1985
121	EF547449.1	mouse	Central African Republic	1974
122	EF547450.1	mouse	Zimbabwe	1986
123	EF547451.1	mouse	South Africa	2003
124	EF547452.1	mouse	South Africa	2006
125	EF547454.1	mouse	South Africa	1980
126	EF547455.1	mouse	South Africa	1982
127	EF547456.1	mouse	South Africa	1980

128	EF547457.1	mouse	South Africa	1980
129	EF547458.1	mouse	South Africa	2004
130	EF547459.1	mouse	Nigeria	1956
131	EU293094.1	Eptesicus serotinus	France	2003
132	EU293095.1	Eidolon helvum	Senegal	1985
133	EU293096.1	shrew	Cameroon	1974
134	EU293097.1	rodent	Central African Republic	1981
135	EU293098.1	Homo sapiens	South Africa	1971
136	EU293099.1	Eidolon helvum	Nigeria	1956
137	EU293101.1	Eptesicus serotinus	France	1989
138	EU293105.1	Miniopterus	South Africa	1981

139	EU352768.1	Eptesicus serotinus	Germany	
140	EU623437.1	Homo sapiens	South Africa	
141	EU623438.1	Chiroptera	South Africa	
142	EU623436.1	Homo sapiens	South Africa	
143	EU623439.1	Chiroptera	South Africa	
144	EU623440.1	Homo sapiens	South Africa	
145	EU623441.1	Chiroptera	South Africa	
146	EU623442.1	Homo sapiens	South Africa	
147	EU623443.1	Chiroptera	South Africa	
148	EU636788.1	Eptesicus serotinus	France	2004
149	EU636789.1	Eptesicus serotinus	France	2005
150	EU636790.1	Eptesicus serotinus	France	2005

151	EU636791.1	Eptesicus serotinus	France	2006
152	EU636792.1	Eptesicus serotinus	France	2006
153	EU636793.1	Eptesicus serotinus	France	2007
154	EU636794.1	Eptesicus serotinus	France	2004
155	FJ465410.1	cat	South Africa	1998
156	FJ465411.1	cat	South Africa	1996
157	FJ465412.1	cat	South Africa	2006
158	FJ465413.1	cat	South Africa	1997
159	FJ465414.1	cat	South Africa	1996
160	FJ465415.1	cat	South Africa	1995
161	FJ465416.1	cat	South Africa	1970
162	FJ465417.1	cat	Zimbabwe	1981

163	FJ465418.1	cat	Zimbabwe	1981
164	GQ500108.1	feline	South Africa	1998
165	GQ500109.1	feline	Zimbabwe	1993
166	GQ500110.1	feline	South Africa	1995
167	GQ500111.1	feline	South Africa	1996
168	GQ500112.1	feline	South Africa	1997
169	GQ500113.1	feline	South Africa	1997
170	GQ500116.1	feline	South Africa	1995
171	GU992303.1	Eptesicus serotinus (bat)	France	2002
172	GU992305.1	Eptesicus serotinus (bat)	France	1989
173	GU992310.1	shrew	Cameroon	1986
174	GU992312.1	bat	Australia	1998

175	GU992313.1	cat	South Africa	1987
176	GU992314.1	rodent	Central African Republic	1986
177	GU992315.1	Homo sapiens	South Africa	1971
178	GQ861350.1	canine	Zimbabwe	1981
179	GQ861351.1	feline	South Africa	2006
180	GQ861352.1	canine	Zimbabwe	1981
181	GQ861353.1	feline	South Africa	1996
182	GQ472989.1	feline	South Africa	1970
183	GQ472990.1	canine	Zimbabwe	1982
184	GQ472991.1	canine	Zimbabwe	1981
185	GQ472993.1	feline	Zimbabwe	1993

186	GQ472995.1	feline	South Africa	1996
187	GQ472996.1	feline	South Africa	1996
188	GQ472997.1	feline	South Africa	1997
189	GQ472998.1	feline	South Africa	1997
190	GQ472999.1	feline	South Africa	2006
191	GQ473000.1	feline	South Africa	1998
192	GQ473001.1	feline	South Africa	
193	GQ473002.1	canine	Zimbabwe	
194	GQ473003.1	canine	Zimbabwe	
195	GQ473004.1	canine	Zimbabwe	
196	HM623779.1	Eidolon helvum	Nigeria	1956
197	HM623780.1	Crocidura sp. (shrew)	Nigeria	1968

198	HM179509.1	Epomophorus wahlbergi	South Africa	2008
199	HQ266623.1	Epomophorus wahlbergi	South Africa	2008
200	HQ266624.1	feline	South Africa	2006
201	HQ266634.1	Epomophorus wahlbergi	South Africa	2008
202	KC567812.1	Eptesicus serotinus	France	2004
203	KC567813.1	Eptesicus serotinus	France	2005
204	KC567814.1	Eptesicus serotinus	France	2009
205	KC567815.1	Eptesicus serotinus	France	2006
206	KC567816.1	Eptesicus serotinus	France	2010
207	KC567817.1	Eptesicus serotinus	France	2009
208	KC567818.1	Eptesicus serotinus	France	2009
209	GQ472992.1	feline	South Africa	1995

210	GQ472994.1	canine	Zimbabwe	1981
211	KF042301.1	Eptesicus serotinus	Germany	2005
212	KF042302.1	Eptesicus serotinus	Germany	1993
213	KC218932.1		Zimbabwe	1981
214	KC218933.1		Zimbabwe	1993
215	KC218934.1		South Africa	2008
216	KC218935.1		South Africa	2008
217	KC218936.1		South Africa	2008
218	KC218937.1		South Africa	2008
219	KP899613.1	domestic cat	South Africa	2012
220	KP899614.1	domestic cat	South Africa	2012
221	KP899615.1	domestic cat	South Africa	2014

222	KP899616.1	domestic cat	South Africa	2012
223	KP899617.1	domestic cat	South Africa	2012
224	KP899618.1	domestic cat	South Africa	2014
225	KP899619.1	domestic cat	South Africa	2012
226	KP899620.1	domestic cat	South Africa	2012
227	KP899621.1	domestic cat	South Africa	2014
228	KU739052.1	Bat	Australia	1998
229	KU739053.1	Homo sapiens	South Africa	1971
230	KU761302.1	Homo sapiens	South Africa	1986
231	KU761303.1	bat	Nigeria	1986
