# Supplementary material for: Comprehensive Analysis of Codon Usage on Rabies Virus and Other Lyssaviruses
Source: Int J Mol Sci. 2018 Aug 14;19(8):2397. doi: 10.3390/ijms19082397 (PMC6121662; doi:10.3390/ijms19082397)
Supplement: Supplementary file 1 [file ijms-19-02397-s001.zip › Table S3 Summary of correlation analysis between several elements in other lyssavirus genome coding sequences..pdf]

Supplemental table 3 Summary of correlation analysis between several elements in other lyssavirus genome coding sequences.

A. Summary of correlation analysis between several elements in LBV genome coding sequences.

	A%	C%	G%	U%	A3s	C3s	G3s	U3s	AU	GC	GC1s	GC2s	GC12s	ENC	Axis1	Axis2	Gravy
**A%**																	
**C%**	**-0.940****																
**G%**	**-0.997****	**0.959****															
**U%**	**0.941****	**-0.999 NS**	**-0.961****														
**A3s**	**0.995****	**-0.903***	**-0.985****	**0.904***													
**C3s**	**-0.959****	**0.997****	**0.973****	**-0.995****	**-0.930****												
**G3s**	**-0.998****	**0.919****	**0.991****	**-0.920****	**-0.998****	**0.942****											
**U3s**	**0.958****	**-0.998****	**-0.974****	**0.998****	**0.926****	**-0.998****	**-0.941****										
**AU**	**0.983****	**-0.986****	**-0.993****	**0.987****	**0.961****	**-0.993****	**-0.971****	**0.993****									
**GC**	**-0.983***	**0.986****	**0.993****	**-0.987****	**-0.961****	**0.993****	**0.971****	**-0.993****	**-1.000****								
**GC1s**	**-0.900NS**	**0.986****	**0.927****	**-0.989****	**-0.852***	**0.973****	**0.872***	**-0.980****	**-0.962****	**0.962****							
**GC2s**	**0.031 NS**	**0.269 NS**	**0.041****	**-0.281 NS**	**0.127 NS**	**0.197 NS**	**-0.090 NS**	**-0.222 NS**	**-0.134 NS**	**0.134 NS**	**0.386 NS**						
**GC12s**	**-0.774****	**0.928****	**0.817***	**-0.933****	**-0.707 NS**	**0.897***	**0.734 NS**	**-0.909***	**-0.871***	**0.871***	**0.970****	**0.598 NS**					
**ENC**	**-0.932****	**0.993****	**0.953****	**-0.995****	**-0.895***	**0.989****	**0.910***	**-0.992****	**-0.979****	**0.979****	**0.979****	**0.304 NS**	**0.931****				
**Axis1**	**-0.995****	**0.965****	**0.997****	**-0.964****	**-0.983****	**0.979****	**0.987****	**-0.976****	**-0.994****	**0.994****	**0.930****	**0.045 NS**	**0.820***	**0.957****			
**Axis2**	**-0.898***	**0.990****	**0.926****	**-0.992****	**-0.854***	**0.980****	**0.875***	**-0.985****	**-0.960****	**0.960****	**0.983****	**0.360 NS**	**0.948****	**0.992****	**0.928****		
**Gravy**	**-0.988****	**0.933****	**0.982****	**-0.930****	**-0.986****	**0.957****	**0.990****	**-0.950****	**-0.972****	**0.972****	**0.877***	**-0.092 NS**	**0.738 NS**	**0.917***	**0.983****	**0.892***	
**Aromatic**	**-0.951****	**0.852***	**0.938****	**-0.850***	**-0.966****	**0.888***	**0.965****	**-0.880****	**-0.909***	**0.909***	**0.769 NS**	**-0.225 NS**	**0.610 NS**	**0.851***	**0.933****	**0.816***	**0.972****

NS means non-significant (P > 0.05).

* represents 0.01 < P < 0.05.

** represents P < 0.01.

B. Summary of correlation analysis between several elements in ABLV genome coding sequences.

	A%	C%	G%	U%	A3s	C3s	G3s	U3s	AU	GC	GC1s	GC2s	GC12s	ENC	Axis1	Axis2	Gravy
**A%**																	
**C%**	**0.277**^{NS}																
**G%**	**-0.551**^{NS}	**-0.931****															
**U%**	**-0.378**^{NS}	**-0.993****	**0.946****														
**A3s**	**0.952****	**0.030**^{NS}	**-0.335**^{NS}	**-0.135**^{NS}													
**C3s**	**0.321**^{NS}	**0.996****	**-0.949****	**-0.992****	**0.088**^{NS}												
**G3s**	**-0.878***	**-0.044**^{NS}	**0.353**^{NS}	**0.130**^{NS}	**-0.963****	**-0.113**^{NS}											
**U3s**	**-0.369**^{NS}	**-0.993****	**0.959****	**0.995****	**-0.137**^{NS}	**-0.999****	**0.155**^{NS}										
**AU**	**-0.267**^{NS}	**-0.990****	**0.893***	**0.989****	**-0.026**^{NS}	**-0.982****	**0.026**^{NS}	**0.980****									
**GC**	**0.267**^{NS}	**0.990****	**-0.893***	**-0.989****	**0.026**^{NS}	**0.982****	**-0.026**^{NS}	**-0.980****	**-1.000****								
**GC1s**	**-0.499**^{NS}	**-0.809**^{NS}	**0.931****	**0.808**	**-0.326**^{NS}	**-0.846***	**0.387**^{NS}	**0.853***	**0.733**^{NS}	**-0.733**^{NS}							
**GC2s**	**0.512**^{NS}	**0.958****	**-0.957****	**-0.982****	**0.261**^{NS}	**0.957****	**-0.221**^{NS}	**-0.968****	**-0.952****	**0.952****	**-0.808**^{NS}						
**GC12s**	**-0.324**^{NS}	**-0.410**^{NS}	**0.603**^{NS}	**0.387**^{NS}	**-0.279**^{NS}	**-0.469**^{NS}	**0.411**^{NS}	**0.470**^{NS}	**0.295**^{NS}	**-0.295**^{NS}	**0.847***	**-0.371**^{NS}					
**ENC**	**0.105**^{NS}	**0.979****	**-0.875***	**-0.948****	**-0.146**^{NS}	**0.969****	**0.117**^{NS}	**-0.956****	**-0.956****	**0.956****	**-0.791**^{NS}	**0.891***	**-0.443**^{NS}				
**Axis1**	**0.460**^{NS}	**0.828***	**-0.789**^{NS}	**-0.863***	**0.290**^{NS}	**0.849***	**-0.274**^{NS}	**-0.863***	**-0.850***	**0.850***	**-0.710**^{NS}	**0.839***	**-0.362**^{NS}	**0.754**^{NS}			
**Axis2**	**0.930****	**0.136**^{NS}	**-0.392**^{NS}	**-0.241**^{NS}	**0.897***	**0.161**^{NS}	**-0.797**^{NS}	**-0.208**^{NS}	**-0.148**^{NS}	**0.148**^{NS}	**-0.253**^{NS}	**0.395**^{NS}	**-0.042**^{NS}	**-0.047**^{NS}	**0.225**^{NS}		
**Gravy**	**-0.289**^{NS}	**0.749**^{NS}	**-0.592**^{NS}	**-0.673**^{NS}	**-0.423**^{NS}	**0.752**^{NS}	**0.272**^{NS}	**-0.718**^{NS}	**-0.717**^{NS}	**0.717**^{NS}	**-0.617**^{NS}	**0.532**^{NS}	**-0.492**^{NS}	**0.837***	**0.530**^{NS}	**-0.468**^{NS}	
**Aromatic**	**0.034**^{NS}	**-0.937****	**0.799**^{NS}	**0.892***	**0.290**^{NS}	**-0.915***	**-0.261**^{NS}	**0.895***	**0.916***	**-0.916***	**0.694**^{NS}	**-0.828***	**0.347**^{NS}	**-0.981****	**-0.627**^{NS}	**0.133**^{NS}	**-0.842***

NS means non-significant ($P > 0.05$).

* represents $0.01 < P < 0.05$.

** represents $P < 0.01$.

C. Summary of correlation analysis between several elements in EBLV genome coding sequences.

	A%	C%	G%	U%	A3s	C3s	G3s	U3s	AU	GC	GC1s	GC2s	GC12s	ENC	Axis1	Axis2	Gravy
A%																	
C%	-0.550**																
G%	-0.882**	0.556*															
U%	0.440NS	-0.928**	-0.626*														
A3s	0.916**	-0.647**	-0.874**	0.584*													
C3s	-0.558*	0.962**	0.621*	-0.939**	-0.713**												
G3s	-0.803**	0.630*	0.928**	-0.696**	-0.905**	0.701**											
U3s	0.490 NS	-0.892**	-0.688**	0.978**	0.625*	-0.939**	-0.750**										
AU	0.798**	-0.851**	-0.887**	0.867**	0.859**	-0.892**	-0.900**	0.910**									
GC	-0.798**	0.851**	0.887**	-0.867**	-0.859**	0.892**	0.900**	-0.910**	-1.000**								
GC1s	0.388 NS	-0.517*	-0.630*	0.671**	0.640*	-0.688**	-0.772**	0.765**	0.723**	-0.723**							
GC2s	-0.514*	0.381 NS	0.723**	-0.525*	-0.448 NS	0.402 NS	0.618*	-0.598*	-0.649**	0.649**	-0.573*						
GC12s	0.187 NS	-0.417 NS	-0.367 NS	0.523*	0.529*	-0.611*	-0.595*	0.597*	0.519*	-0.519*	0.895**	-0.146 NS					
ENC	-0.207 NS	0.411 NS	0.320 NS	-0.468 NS	-0.140 NS	0.412 NS	0.168 NS	-0.502**	-0.450 NS	0.450 NS	-0.299 NS	0.657**	-0.003 NS				
Axis1	-0.622*	0.668**	0.713**	-0.697**	-0.852**	0.787**	0.821**	-0.726 NS	-0.790**	0.790**	-0.816**	0.388 NS	-0.773**	0.098 NS			
Axis2	0.288 NS	-0.227 NS	-0.402 NS	0.305 NS	0.447 NS	-0.361 NS	-0.426 NS	0.392 NS	0.426 NS	-0.426 NS	0.725**	-0.597*	0.550*	-0.480 NS	-0.567*		
Gravy	-0.282 NS	0.235 NS	0.432 NS	-0.339 NS	-0.310 NS	0.380 NS	0.380 NS	-0.481 NS	-0.472 NS	0.472 NS	-0.599*	0.559*	-0.418 NS	0.597*	0.306 NS	-0.604*	
Aromatic	0.452 NS	-0.670**	-0.545*	0.694**	0.489 NS	-0.691**	-0.449 NS	0.701**	0.688**	-0.688**	0.498 NS	-0.633*	0.255 NS	-0.686**	-0.594*	0.652**	-0.491 NS

NS means non-significant ($P > 0.05$).

* represents $0.01 < P < 0.05$.

** represents $P < 0.01$.

D. Summary of correlation analysis between several elements in DUVV genome coding sequences.

	A%	C%	G%	U%	A3s	C3s	G3s	U3s	AU	GC	GC1s	GC2s	GC12s	ENC	Axis1	Axis2	Gravy
**A%**																	
**C%**	-0.994**																
**G%**	-0.985*	0.992**															
**U%**	0.920^{NS}	-0.949^{NS}	-0.973*														
**A3s**	0.993**	-0.975*	-0.969*	0.887^{NS}													
**C3s**	-0.972*	0.969*	0.930^{NS}	-0.846^{NS}	-0.957*												
**G3s**	-0.976*	0.976*	0.995**	-0.965*	-0.970*	0.901^{NS}											
**U3s**	0.881^{NS}	-0.923^{NS}	-0.944^{NS}	0.993**	0.834^{NS}	-0.817^{NS}	-0.927^{NS}										
**AU**	0.998**	-0.992**	-0.991**	0.933^{NS}	0.993**	-0.957*	-0.987*	0.893^{NS}									
**GC**	-0.998**	0.992**	0.991*	-0.933^{NS}	-0.993**	0.957*	0.987*	-0.893^{NS}	-1.000**								
**GC1s**	-0.997**	0.999**	0.987^{NS}	-0.934^{NS}	-0.981*	0.977*	0.972*	-0.904^{NS}	-0.994**	0.994**							
**GC2s**	-0.758^{NS}	0.826^{NS}	0.829*	-0.912^{NS}	-0.682^{NS}	0.739^{NS}	0.786^{NS}	-0.954*	-0.762^{NS}	0.762^{NS}	0.802^{NS}						
**GC12s**	-0.955*	0.982*	0.976**	-0.972*	-0.916^{NS}	0.935^{NS}	0.949*	-0.968*	-0.955*	0.955*	0.974*	0.916^{NS}					
**ENC**	-0.985*	0.996**	0.998**	-0.973*	-0.963*	0.944^{NS}	0.985*	-0.950*	-0.988*	0.988*	0.991**	0.853^{NS}	0.988*				
**Axis1**	-0.993**	0.992**	0.997**	-0.953*	-0.985*	0.942^{NS}	0.994**	-0.916^{NS}	-0.998**	0.998**	0.991**	0.787^{NS}	0.962*	0.993**			
**Axis2**	-0.989*	0.997**	0.998**	-0.968*	-0.970*	0.947^{NS}	0.987*	-0.942^{NS}	-0.992**	0.992**	0.993**	0.839^{NS}	0.984*	1.000**	0.996**		
**Gravy**	-0.969*	0.990**	0.977*	-0.957*	-0.935^{NS}	0.958*	0.950*	-0.947^{NS}	-0.966*	0.966*	0.985*	0.889^{NS}	0.997**	0.989*	0.969*	0.987*	
**Aromatic**	0.933^{NS}	-0.959*	-0.980*	0.999**	0.902^{NS}	-0.863^{NS}	-0.972*	0.989*	0.945^{NS}	-0.945^{NS}	-0.946^{NS}	-0.905^{NS}	-0.977*	-0.980*	-0.963*	-0.976*	-0.964*

NS means non-significant ($P > 0.05$).

* represents $0.01 < P < 0.05$.

** represents $P < 0.01$.

E. Summary of correlation analysis between several elements in MOKV genome coding sequences.

	A%	C%	G%	U%	A3s	C3s	G3s	U3s	AU	GC	GC1s	GC2s	GC12s	ENC	Axis1	Axis2	Gravy
**A%**																	
**C%**	**-0.184** ^{NS}																
**G%**	**-0.851** *	**0.165** ^{NS}															
**U%**	**0.059** ^{NS}	**-0.901** *	**-0.278** ^{NS}														
**A3s**	**0.906** *	**-0.008** ^{NS}	**-0.970** **	**0.067** ^{NS}													
**C3s**	**0.012** ^{NS}	**0.935** **	**-0.136** ^{NS}	**-0.756** ^{NS}	**0.257** ^{NS}												
**G3s**	**-0.847** *	**0.113** ^{NS}	**0.998** **	**-0.232** ^{NS}	**-0.980** **	**-0.182** ^{NS}											
**U3s**	**-0.010** ^{NS}	**-0.966** **	**0.082** ^{NS}	**0.825** *	**-0.230** ^{NS}	**-0.988** **	**0.134** ^{NS}										
**AU**	**0.671** ^{NS}	**-0.771** ^{NS}	**-0.755** ^{NS}	**0.779** ^{NS}	**0.632** ^{NS}	**-0.535** ^{NS}	**-0.719** ^{NS}	**0.589** ^{NS}									
**GC**	**-0.671** ^{NS}	**0.771** ^{NS}	**0.755** ^{NS}	**-0.779** ^{NS}	**-0.632** ^{NS}	**0.535** ^{NS}	**0.719** ^{NS}	**-0.589** ^{NS}	**-1.000** **								
**GC1s**	**-0.217** ^{NS}	**-0.079** ^{NS}	**0.648** ^{NS}	**-0.275** ^{NS}	**-0.581** ^{NS}	**-0.289** ^{NS}	**0.672** ^{NS}	**0.250** ^{NS}	**-0.372** ^{NS}	**0.372** ^{NS}							
**GC2s**	**-0.246** ^{NS}	**0.302** ^{NS}	**-0.009** ^{NS}	**-0.075** ^{NS}	**0.030** ^{NS}	**0.227** ^{NS}	**-0.056** ^{NS}	**-0.290** ^{NS}	**-0.185** ^{NS}	**0.185** ^{NS}	**-0.673** ^{NS}						
**GC12s**	**-0.551** ^{NS}	**0.217** ^{NS}	**0.854** *	**-0.446** ^{NS}	**-0.742** ^{NS}	**-0.142** ^{NS}	**0.836** *	**0.024** ^{NS}	**-0.693** *	**0.693** *	**0.614** ^{NS}	**0.171** ^{NS}					
**ENC**	**-0.838** *	**0.639** ^{NS}	**0.660** ^{NS}	**-0.451** ^{NS}	**-0.623** ^{NS}	**0.462** ^{NS}	**0.622** ^{NS}	**-0.492** ^{NS}	**-0.848** ^{NS}	**0.848** ^{NS}	**-0.054** ^{NS}	**0.544** ^{NS}	**0.508** ^{NS}				
**Axis1**	**-0.520** ^{NS}	**0.793** ^{NS}	**0.340** ^{NS}	**-0.586** ^{NS}	**-0.250** ^{NS}	**0.661** ^{NS}	**0.283** ^{NS}	**-0.713** ^{NS}	**-0.741** ^{NS}	**0.741** ^{NS}	**-0.324** ^{NS}	**0.756** ^{NS}	**0.375** ^{NS}	**0.895** *			
**Axis2**	**-0.082** ^{NS}	**0.696** ^{NS}	**0.224** ^{NS}	**-0.751** ^{NS}	**-0.109** ^{NS}	**0.687** ^{NS}	**0.213** ^{NS}	**-0.662** ^{NS}	**-0.614** ^{NS}	**0.614** ^{NS}	**0.438** ^{NS}	**-0.467** ^{NS}	**0.086** ^{NS}	**0.251** ^{NS}	**0.196** ^{NS}		
**Gravy**	**-0.285** ^{NS}	**0.269** ^{NS}	**-0.157** ^{NS}	**0.104** ^{NS}	**0.045** ^{NS}	**0.487** ^{NS}	**-0.155** ^{NS}	**-0.362** ^{NS}	**-0.081** ^{NS}	**0.081** ^{NS}	**-0.450** ^{NS}	**0.016** ^{NS}	**-0.581** ^{NS}	**0.337** ^{NS}	**0.247** ^{NS}	**0.303** ^{NS}	
**Aromatic**	**-0.091** ^{NS}	**0.360** ^{NS}	**-0.214** ^{NS}	**-0.086** ^{NS}	**0.164** ^{NS}	**0.595** ^{NS}	**-0.216** ^{NS}	**-0.477** ^{NS}	**-0.104** ^{NS}	**0.104** ^{NS}	**-0.279** ^{NS}	**-0.215** ^{NS}	**-0.600** ^{NS}	**0.197** ^{NS}	**0.134** ^{NS}	**0.542** ^{NS}	**0.924** **

NS means non-significant ($P > 0.05$).

* represents $0.01 < P < 0.05$.

** represents $P < 0.01$.
