# Supplementary material for: Comprehensive Analysis of Codon Usage on Rabies Virus and Other Lyssaviruses
Source: Int J Mol Sci. 2018 Aug 14;19(8):2397. doi: 10.3390/ijms19082397 (PMC6121662; doi:10.3390/ijms19082397)
Supplement: Supplementary file 1 [file ijms-19-02397-s001.zip › Table S4 Characteristic of lyssavirus genomes analyzed in present study..pdf]

Supplemental table 4 Characteristic of lyssavirus genomes analyzed in present study.

A. Characteristic of RABV genomes analyzed in present study.

No	Accession	Name	Host	Country	Year
1	AB569299	H-08-1320	Homo sapiens	Sri Lanka	2008
2	AB635373	H-1413-09	Paradoxurus zeylonensis	Sri Lanka	2009
3	EF437215	NNV-RAB-H	Homo sapiens	India	—
4	EU293113	9001FRA	dog	Guyana	1990
5	EU293115	9147FRA	fox	France	1991
6	EU293116	9704ARG	Tadarida brasiliensis	Argentina	1997
7	EU886632	2002 from Germany	red fox	Germany	2002
8	EU886633	2004 from Austria	red fox	Austria	2004
9	EU886634	2004 from Germany	red fox	Germany	2004
10	EU886635	2005 from Germany	red fox	Germany	2005
11	EU886636	2006 from Austria	red fox	Austria	2006
12	FJ866835	FJ008	dog	China: Fujian	2008
13	FJ866836	FJ009	dog	China: Fujian	2008
14	GU345746	CQ92	dog	China: Chongqing	1992
15	GU345747	J	Homo sapiens	China: Ningxia	1986
16	GU345748	SH06	dog	China: Shanghai	2006
17	HE802675	Pk23	Bos taurus	Pakistan	2010
18	HQ450385	DRV-AH08	dog	China	2008
19	JN234411	4aGV	dog	China	1931
20	JN609295	FJDRV	Canis lupus familiaris	China	2008
21	JQ423952	BJ2011E	equine	China	2011
22	JQ647510	WH11	donkey	China	2011

23	JQ685892	2398	gray fox	USA	2009
24	JQ685893	SM5079	striped skunk	USA	2001
25	JQ685894	CA982	striped skunk	USA	1994
26	JQ685895	WA1185	Lasionycteris noctivagans	USA	2003
27	JQ685896	2402	gray fox	USA	2009
28	JQ685897	SM5442	Eptesicus fuscus	USA	2001
29	JQ685898	A093500	Eptesicus fuscus	USA	2009
30	JQ685899	A10-0515	gray fox	USA	2009
31	JQ685900	FL769	Lasiurus seminolus	USA	2003
32	JQ685902	TN209	Lasiurus borealis	USA	2005
33	JQ685903	CA04148	Eptesicus fuscus	USA	2004
34	JQ685904	SM5081	striped skunk	USA	2001
35	JQ685905	FL385	Tadarida brasiliensis	USA	2003
36	JQ685906	SM5102	striped skunk	USA	2001
37	JQ685907	SM3849	Eptesicus fuscus	USA	1996
38	JQ685909	CA100	Eptesicus fuscus	USA	2005
39	JQ685910	TX5960	Lasiurus xanthinus	USA	2002
40	JQ685911	SM5077	striped skunk	USA	2001
41	JQ685912	2400	gray fox	USA	2009
42	JQ685913	AZBAT-6763	Eptesicus fuscus	USA	1985
43	JQ685914	OR703	gray fox	USA	2010
44	JQ685915	TX4904	Lasiurus intermedius	USA	2002
45	JQ685916	FL1010	Lasiurus intermedius	USA	2002
46	JQ685917	CO-Coyot-2010	coyote	USA	2010
47	JQ685918	OR05506	gray fox	USA	2010

48	JQ685919	NJ2262	Lasiurus borealis	USA	2005
49	JQ685920	EF	Eptesicus fuscus	USA	1984
50	JQ685921	FL1078	Myotis austroriparius	USA	2001
51	JQ685922	TN186	Perimyotis subflavus	USA	2005
52	JQ685923	SM4871	Eptesicus fuscus	USA	1999
53	JQ685925	WAEF03	Eptesicus fuscus	USA	2004
54	JQ685926	AZ2408	Eptesicus fuscus	USA	2005
55	JQ685927	SM5080	striped skunk	USA	2001
56	JQ685928	2403	gray fox	USA	2009
57	JQ685929	MEXSK3644	Spilogale putorius (Spotted skunk)	Mexico	2009
58	JQ685930	SM5103	striped skunk	USA	2001
59	JQ685931	WA0173	Eptesicus fuscus	USA	2000
60	JQ685932	SM5076	striped skunk	USA	2001
61	JQ685933	SM5950	gray fox	USA	2004
62	JQ685934	2401	gray fox	USA	2009
63	JQ685936	3634DR	bovine	Mexico	2009
64	JQ685937	2396	Bassariscus astutus	USA	2009
65	JQ685938	A10-0514	striped skunk	USA	2009
66	JQ685939	2399	gray fox	USA	2009
67	JQ685940	SM5100	striped skunk	USA	2001
68	JQ685941	SM1545	striped skunk	USA	2005
69	JQ685942	AZBAT-65094	Eptesicus fuscus	USA	1981
70	JQ685943	A10-0511	gray fox	USA	2009
71	JQ685944	NC839	striped skunk	USA	1984
72	JQ685945	SM6709	domestic cat (Felis catus)	USA	2005

73	JQ685946	SM4862	Eptesicus fuscus	USA	1999
74	JQ685947	TN310	Lasiurus cinereus	USA	2004
75	JQ685948	OR05455	gray fox	USA	2010
76	JQ685949	SM5075	striped skunk	USA	2001
77	JQ685950	A093504	Eptesicus fuscus	USA	2009
78	JQ685951	AZ10-144	Eptesicus fuscus	USA	2010
79	JQ685952	A02-2971	Parastrellus hesperus	USA	2002
80	JQ685953	3645DR	Homo sapiens	Mexico	2009
81	JQ685954	MEXSK13938	Spilogale putorius (Spotted skunk)	Mexico	2007
82	JQ685955	AZ4490	Myotis yumanensis	USA	2005
83	JQ685956	AZBAT-7453	Eptesicus fuscus	USA	1975
84	JQ685957	OR8767	gray fox	USA	2009
85	JQ685958	SM5101	striped skunk	USA	2001
86	JQ685959	SM5451	striped skunk	USA	2001
87	JQ685960	SM4872	Eptesicus fuscus	USA	2001
88	JQ685961	AZ10-140	Eptesicus fuscus	USA	2010
89	JQ685962	SM5441	striped skunk	USA	2001
90	JQ685963	Coati-3639	Nasua narica (coati)	Mexico	2009
91	JQ685964	SM5596	striped skunk	USA	2004
92	JQ685965	A02-2972	Parastrellus hesperus	USA	2002
93	JQ685966	SM5470	striped skunk	USA	2001
94	JQ685968	A10-0512	striped skunk	USA	2009
95	JQ685970	CASK2	striped skunk	USA	1974
96	JQ685971	AZ3003	Antrozous pallidus	USA	2009
97	JQ685972	2395	gray fox	USA	2009

98	JQ685973	A11-1043	coyote	USA	2011
99	JQ685974	SM3844	Eptesicus fuscus	USA	1995
100	JQ685975	MEXSK3636	Spilogale putorius (Spotted skunk)	Mexico	2009
101	JQ685976	2526	gray fox	USA	2009
102	JQ944704	184VNO	raccoon dog	Russia	2009
103	JQ944705	1350KRA	dog	Russia	2008
104	JQ944706	1352KRA	dog	Russia	2008
105	JQ944707	1410KOM	deer	Russia	2008
106	JQ944708	1564NNO	red fox	Russia	2008
107	JQ970481	CJS0621D	dog	China	2006
108	JQ970482	CJS0636D	dog	China	2006
109	JQ970483	CJS0848D	dog	China	2008
110	JQ970484	CJX0903D	dog	China	2009
111	JQ970485	CJX0906D	dog	China	2009
112	JQ970486	CSD0708D	dog	China	2007
113	JQ970487	CSX0904D	dog	China	2009
114	JX473838	178J09	jackal	Namibia	2009
115	JX473839	192J09	jackal	Namibia	2009
116	JX473840	239K09	Tragelaphus strepsiceros (Namibian kudu)	Namibia	2009
117	JX473841	240K09	Tragelaphus strepsiceros (Namibian kudu)	Namibia	2009
118	KC169986	GXHXN	rabid cattle	China	2009
119	KC171644	BD0406CC	raccoon dog	South Korea	2004
120	KC171645	BV9901PJ	raccoon dog	South Korea	1999

121	KC193267	CNM1101C	cow	China	2011
122	KC196743	DRV-NG11	dog	Nigeria	2011
123	KC252633	CNM1103C	bovine	China	2011
124	KC252634	CNM1104D	dog	China	2011
125	KC595280	Rus(Lipetsk)8052f_2011	Vulpes vulpes (red fox)	Russia	2011
126	KC595281	Rus(Lipetsk)8053c_2011	cat	Russia	2011
127	KC595282	Rus(Lipetsk)8054f_2011	Vulpes vulpes (red fox)	Russia	2011
128	KC595283	Rus(Lipetsk)8057f_2011	Vulpes vulpes (red fox)	Russia	2011
129	KC737850	A11-5300	Homo sapiens	USA	2011
130	KC762941	JX09-17(fb)	Melogale moschata	China	2009
131	KC977995	Shaanxi-HZ-6	canine	China	2009
132	KF154996	RV61	Homo sapiens	United Kingdom	1987
133	KF154997	RV437	raccoon dog	Estonia	—
134	KF154998	RV2324	dog	Israel	1950
135	KF154999	RV2417	dog	United Kingdom	2008
136	KF155000	RV2516	cow	Iraq	2010
137	KF155001	RV2627	cow	Morocco	2009
138	KF155002	RV2772	dog	Tanzania	2010
139	KF620487	R2012-26	ferret badger	Taiwan	2012
140	KF620489	R2013-01	ferret badger	Taiwan	2013
141	KF726853	JX10-37	Melogale moschata (ferret badger)	China	2010
142	KF977826	CAR_11/001h	Homo sapiens	Central African Republic	2011
143	KJ004416	JZ13-Lv	Homo sapiens	China	2013
144	KJ564280	IMDRV-13	Dama dama (fallow deer)	China	2013

145	KM016899	WQ14-RF	red fox	China	2014
146	KM272192	CQH1202D/China/2012	dog	China	2012
147	KM594023	IP6634/08	Callithrix jacchus	Brazil	2008
148	KM594025	IP5402/07	Callithrix jacchus	Brazil	2007
149	KM594026	IP512/09	Eptesicus furinalis	Brazil	2009
150	KM594027	IP230/10	Eptesicus furinalis	Brazil	2010
151	KM594028	IP346/10	Eptesicus furinalis	Brazil	2010
152	KM594029	IP3208/06	Eptesicus furinalis	Brazil	2006
153	KM594030	IP1400/10	Myotis nigricans	Brazil	2010
154	KM594031	IP163/10	Myotis nigricans	Brazil	2010
155	KM594032	IP497/10	Myotis nigricans	Brazil	2010
156	KM594033	IP4431/10	Tadarida brasiliensis	Brazil	2010
157	KM594034	IP350/10	Nyctinomops laticaudatus	Brazil	2010
158	KM594035	IP412/10	Nyctinomops laticaudatus	Brazil	2010
159	KM594036	IP542/10	Nyctinomops laticaudatus	Brazil	2010
160	KM594037	IP3176/09	Tadarida brasiliensis	Brazil	2009
161	KM594038	IP1586/10	Tadarida brasiliensis	Brazil	2010
162	KM594039	IP7841/09	Cerdocyon thous	Brazil	2009
163	KM594040	IP2992/13	Desmodus rotundus	Brazil	2013
164	KM594041	IP2990/13	Desmodus rotundus	Brazil	2013
165	KM594042	IP2991/13	Desmodus rotundus	Brazil	2013
166	KM594043	IP4005/12	Bos taurus	Brazil	2012
167	KP723638	RV2985	Canis simensis	Ethiopia	2014
168	KP997032	PO-01_2014_Primorye	Ursus arctos	Russia	2014
169	KR230089	SXBJ15	cattle	China	2015

170	KR230090	SXYL15	cattle	China	2015
171	KR534217	RV2490	domestic dog	Tanzania	2008
172	KR534218	RV2491	domestic dog	Tanzania	2008
173	KR534219	RV2492	domestic dog	Tanzania	2007
174	KR534232	RV2792	jackal	Tanzania	2011
175	KR534233	RV2794	domestic dog	Tanzania	2011
176	KR534234	RV2795	cow	Tanzania	2011
177	KR534235	RV2796	cow	Tanzania	2011
178	KR534236	RV2797	cow	Tanzania	2011
179	KR534245	RV2890	cow	Tanzania	2011
180	KR534247	RV2892	domestic dog	Tanzania	2011
181	KR534248	RV2893	cow	Tanzania	2011
182	KR534250	RV2897	domestic dog	Tanzania	2011
183	KR534251	RV2898	domestic dog	Tanzania	2011
184	KR534252	RV2899	domestic dog	Tanzania	2011
185	KR534254	RV2906	cat	Tanzania	2011
186	KR906734	RV2495	domestic dog	Tanzania	2008
187	KR906735	RV2498	domestic dog	Tanzania	2008
188	KR906736	RV2499	domestic dog	Tanzania	2008
189	KR906738	RV2501	cow	Tanzania	2004
190	KR906739	RV2502	domestic dog	Tanzania	2004
191	KR906740	RV2503	wild cat	Tanzania	2009
192	KR906741	RV2504	domestic dog	Tanzania	2003
193	KR906742	RV2767	domestic dog	Tanzania	2009
194	KR906743	RV2770	domestic dog	Tanzania	2010

195	KR906744	RV2771	domestic dog	Tanzania	2010
196	KR906745	RV2773	domestic dog	Tanzania	2010
197	KR906746	RV2774	domestic dog	Tanzania	2010
198	KR906747	RV2775	domestic dog	Tanzania	2010
199	KR906749	RV2777	domestic dog	Tanzania	2010
200	KR906750	RV2778	domestic dog	Tanzania	2010
201	KR906751	RV2780	domestic dog	Tanzania	2011
202	KR906752	RV2782	domestic dog	Tanzania	2011
203	KR906753	RV2783	cow	Tanzania	2011
204	KR906754	RV2784	domestic dog	Tanzania	2011
205	KR906755	RV2793	domestic dog	Tanzania	2011
206	KR906756	RV2799	domestic dog	Tanzania	2011
207	KR906760	RV2810	domestic dog	Tanzania	2010
208	KR906762	RV2813	domestic dog	Tanzania	2010
209	KR906763	RV2814	domestic dog	Tanzania	2010
210	KR906765	RV2816	domestic dog	Tanzania	2010
211	KR906766	RV2817	domestic dog	Tanzania	2010
212	KR906767	RV2861	domestic dog	Tanzania	2011
213	KR906768	RV2862	domestic dog	Tanzania	2011
214	KR906770	RV2875	domestic dog	Tanzania	2011
215	KR906771	RV2894	domestic dog	Tanzania	2011
216	KR906772	RV2896	domestic dog	Tanzania	2011
217	KR906773	RV2900	domestic dog	Tanzania	2011
218	KR906776	RV2907	domestic dog	Tanzania	2011
219	KR906777	RV3091	unknown	Tanzania	2011

220	KR906778	RV3093	unknown	Tanzania	2011
221	KR906780	RV3104	domestic dog	Tanzania	2011
222	KR906781	RV3107	unknown	Tanzania	2012
223	KR906782	RV3111	domestic dog	Tanzania	2012
224	KR906784	RV3125	domestic dog	Tanzania	2012
225	KR906785	RV3127	unknown	Tanzania	2012
226	KR906787	RV3131	cow	Tanzania	2012
227	KR906788	RV3132	cow	Tanzania	2012
228	KR906789	RV3133	domestic dog	Tanzania	2012
229	KR906790	RV3140	domestic dog	Tanzania	2012
230	KR906792	RV3123	domestic dog	Tanzania	2012
231	KT006769	InDRE3944	dog	Mexico	2013
232	KT336432	55612	Canis lupus familiaris	South Africa	2012
233	KT336433	20034	Canis lupus familiaris	Zimbabwe	1991
234	KT336434	21057	Canis lupus familiaris	Zimbabwe	1992
235	KT336435	21467	Canis lupus familiaris	Zimbabwe	1993
236	KT336436	33512	Canis lupus familiaris	South Africa	2012
237	KT336437	34312	Canis lupus familiaris	South Africa	2012
238	KT728348	Rus(Astrakhan)_8329H_2003	Homo sapiens	Russia	2003
239	KT728349	Rus(Astrakhan)_8330H_2003	Homo sapiens	Russia	2003
240	KU198460	89V809AFX	Alopex lagopus	USA	1989
241	KU198461	90L2968AFX	Alopex lagopus	Canada	1990
242	KU198462	90V814AFX	Alopex lagopus	USA	1990
243	KU198463	90V820RFX	Vulpes vulpes	USA	1990
244	KU198464	91L0085AFX	Alopex lagopus	Canada	1991

245	KU198465	92N7894RFX	Vulpes vulpes	Canada	1992
246	KU198466	93N1395AFX	Alopex lagopus	Canada	1993
247	KU198467	97L1793AFX	Alopex lagopus	Canada	1997
248	KU198468	00N3340RFX	Vulpes vulpes	Canada	2000
249	KU198469	01N10254RFX	Vulpes vulpes	Canada	2001
250	KU198470	02N2980RFX	Vulpes vulpes	Canada	2002
251	KU198471	07V1483RFX	Vulpes vulpes	USA	2007
252	KU198472	12L1020AFX	Alopex lagopus	Canada	2012
253	KU198473	12N0215RFX	Vulpes vulpes	Canada	2012
254	KU198474	12N0280RFX	Vulpes vulpes	Canada	2012
255	KU198475	13L0040AFX	Alopex lagopus	Canada	2013
256	KU198476	13L0094AFX	Alopex lagopus	Canada	2013
257	KU198477	13N0473AFX	Alopex lagopus	Canada	2013
258	KU198478	13NO643AFX	Alopex lagopus	Canada	2013
259	KU198479	14N0601RFX	Vulpes vulpes	Canada	2014
260	KU523255	AT6	Desmodus rotundus	French Guiana	2010
261	KX036361	RV1391-GRL-1-F-1990	Arctic fox	Greenland	1990
262	KX036363	RV1416-GRL-5-AF-2002	Arctic fox	Greenland	2002
263	KX036364	RV1417-GRL-3-AF-2002	Arctic fox	Greenland	2002
264	KX036365	RV1418-GRL-4-AF-2002	Arctic fox	Greenland	2002
265	KX036366	RV1419-GRL-3-AF-2002	Arctic fox	Greenland	2002
266	KX036367	RV1420-GRL-4-AF-2002	Arctic fox	Greenland	2002
267	KX148100	09035FRA	Stenodermatinae sp.	French Guiana	2009
268	KX148101	86092EGY	Homo sapiens	Egypt	1979
269	KX148102	91026MEX	dog	Mexico	1991

270	KX148103	87021AFS	Homo sapiens	South Africa	1981
271	KX148104	86058YOU	cow	Montenegro	1978
272	KX148105	86083GRO	dog	Greenland	1980
273	KX148106	91005USA	fox	USA	1990
274	KX148107	86097BEN	cat	Benin	1986
275	KX148108	11001NEP	cattle	Nepal	2011
276	KX148109	86117BRE	dog	Brazil	1986
277	KX148110	91014MEX	dog	Mexico	1991
278	KX148111	91015MEX	dog	Mexico	1991
279	KX148112	91010MEX	Homo sapiens	Mexico	1991
280	KX148113	92002GER	fox	Germany	1991
281	KX148114	96104POL	fox	Poland	1995
282	KX148115	96250POL	fox	Poland	1996
283	KX148116	96256POL	fox	Poland	1996
284	KX148117	97046POL	fox	Poland	1997
285	KX148118	96077POL	raccoon dog	Poland	1994
286	KX148119	96045POL	fox	Poland	1993
287	KX148120	96140POL	raccoon dog	Poland	1993
288	KX148121	96056POL	fox	Poland	1993
289	KX148122	92012GER	fox	Germany	1991
290	KX148123	92017GER	fox	Germany	1991
291	KX148124	92010GER	fox	Germany	1991
292	KX148125	94033BEL	fox	Belgium	1994
293	KX148126	96002FRA	fox	France	1996
294	KX148127	91047FRA	fox	France	1991

295	KX148128	92044FRA	fox	France	1992
296	KX148129	94091SLN	fox	Slovenia	1994
297	KX148130	94096SLN	fox	Slovenia	1994
298	KX148131	94086SLN	fox	Slovenia	1994
299	KX148132	94094SLN	fox	Slovenia	1994
300	KX148133	86111YOU	fox	Bosnia and Herzegovina	1986
301	KX148134	94029FRA	fox	France	1994
302	KX148135	92001GER	fox	Germany	1991
303	KX148136	93083HON	fox	Hungary	1993
304	KX148137	93089HON	fox	Hungary	1993
305	KX148138	93091HON	fox	Hungary	1993
306	KX148139	93093HON	fox	Hungary	1993
307	KX148140	86109YOU	fox	Serbia	1986
308	KX148141	96135POL	fox	Poland	1992
309	KX148142	96177POL	fox	Poland	1994
310	KX148143	93080HON	fox	Hungary	1993
311	KX148145	86054YOU	wolf	Bosnia and Herzegovina	1986
312	KX148146	93046FIN	fox	Finland	1993
313	KX148147	93048FIN	raccoon dog	Finland	1988
314	KX148148	93038EST	fox	Estonia	1992
315	KX148149	93039EST	raccoon dog	Estonia	1991
316	KX148150	96026POL	raccoon dog	Poland	1986
317	KX148151	96042POL	fox	Poland	1996

318	KX148152	96142POL	raccoon dog	Poland	1996
319	KX148153	96097POL	raccoon dog	Poland	1996
320	KX148154	97078POL	raccoon dog	Poland	1997
321	KX148155	93041EST	fox	Estonia	1993
322	KX148156	93042EST	raccoon dog	Estonia	1991
323	KX148157	93043EST	raccoon dog	Estonia	1993
324	KX148158	93105EST	fox	Estonia	1993
325	KX148159	86082IRA	sheep	Iran	1974
326	KX148160	92015HON	Homo sapiens	Hungary	1991
327	KX148161	86133SER	fox	Serbia	1972
328	KX148162	94002TUR	dog	Turkey	1993
329	KX148163	94016TUR	dog	Turkey	1993
330	KX148164	93100TUR	dog	Turkey	1993
331	KX148165	94009TUR	dog	Turkey	1993
332	KX148166	94001TUR	dog	Turkey	1993
333	KX148167	94005TUR	dog	Turkey	1993
334	KX148168	87006ARS	fox	Saudi Arabia	1987
335	KX148169	91034OMA	camel	Oman	1990
336	KX148170	91035OMA	fox	Oman	1990
337	KX148171	91024EAU	fox	United Arab Emirates	1991
338	KX148172	87007ARS	horse	Saudi Arabia	1987
339	KX148173	97139ARS	fox	Saudi Arabia	1997
340	KX148174	98001ARS	fox	Saudi Arabia	1998
341	KX148175	99004ARS	fox	Saudi Arabia	1998
342	KX148176	04034ARS	fox	Saudi Arabia	2004

343	KX148177	96317ISR	fox	Israel	1996
344	KX148178	96306ISR	dog	Israel	1996
345	KX148179	96314ISR	jackal	Israel	1996
346	KX148180	93030ISR	fox	Israel	1993
347	KX148181	96312ISR	dog	Israel	1996
348	KX148182	96307ISR	dog	Israel	1996
349	KX148183	93031ISR	fox	Israel	1993
350	KX148184	96324ISR	cattle	Israel	1996
351	KX148185	96299IRA	wolf	Iran	1996
352	KX148186	87002IRA	wolf	Iran	1984
353	KX148187	96320IRA	jackal	Iran	1996
354	KX148188	93009IRA	wolf	Iran	1991
355	KX148189	86081IRA	dog	Iran	1985
356	KX148190	93008IRA	jackal	Iran	1976
357	KX148191	93032ISR	jackal	Israel	1993
358	KX148192	93033ISR	dog	Israel	1993
359	KX148198	93128SOM	dog	Somalia	1993
360	KX148199	93006SOM	jackal	Somalia	1993
361	KX148200	88008ETH	dog	Ethiopia	1988
362	KX148202	95049GAB	dog	Gabon	1995
363	KX148203	86031MOZ	dog	Mozambique	1986
364	KX148204	92030NAM	dog	Namibia	1992
365	KX148205	94289RWA	dog	Rwanda	1994
366	KX148206	96013TAN	dog	Tanzania	1996
367	KX148207	14015ITA	Homo sapiens	Kenya	2014

368	KX148208	92029CAR	dog	Central African Republic	1992
369	KX148209	04033MAD	dog	Madagascar	2004
370	KX148210	98002MAD	Homo sapiens	Madagascar	1998
371	KX148211	86046MAD	dog	Madagascar	1986
372	KX148212	93019IRA	jackal	Iran	1993
373	KX148213	91001USA	skunk	USA	1982
374	KX148214	95023BRE	dog	Brazil	1995
375	KX148215	95022BRE	dog	Brazil	1995
376	KX148216	86001BRE	dog	Brazil	1986
377	KX148217	86123BRE	dog	Brazil	1986
378	KX148218	14016BOT	wild cat	Botswana	2009
379	KX148219	14017BOT	honey badger	Botswana	2009
380	KX148220	15001AFS	mongoose	South Africa	2013
381	KX148221	15003AFS	squirrel	South Africa	2014
382	KX148222	15002AFS	mongoose	South Africa	2014
383	KX148223	14018AFS	feline	South Africa	2000
384	KX148224	91004USA	skunk	USA	1991
385	KX148225	02052AFG	dog	Afghanistan	2002
386	KX148226	04035AFG	dog	Afghanistan	2002
387	KX148227	04029AFG	dog	Afghanistan	2004
388	KX148228	99001NEP	dog	Nepal	1998
389	KX148229	90012NIG	dog	Niger	1990
390	KX148230	95047HAV	dog	Burkina Faso	1995
391	KX148231	90010NIG	dog	Niger	1990

392	KX148232	92037CI	dog	Cote d'Ivoire	1992
393	KX148233	92038CI	dog	Cote d'Ivoire	1992
394	KX148234	86036HAV	dog	Burkina Faso	1986
395	KX148235	01007CI	dog	Cote d'Ivoire	2001
396	KX148236	93011MAU	dog	Mauritania	1993
397	KX148237	93012MAU	dog	Mauritania	1993
398	KX148238	93003SEN	dog	Senegal	1991
399	KX148239	93005SEN	dog	Senegal	1992
400	KX148240	90021TCH	dog	Chad	1990
401	KX148241	96009TCH	dog	Chad	1996
402	KX148242	95002CAM	dog	Cameroon	1994
403	KX148243	88003CAM	dog	Cameroon	1987
404	KX148244	90024GUI	dog	Guinea	1990
405	KX148245	09029NEP	buffalo	Nepal	2009
406	KX148246	97002IND	Homo sapiens	India	1997
407	KX148247	99009BIR	dog	Myanmar	1999
408	KX148248	99015BIR	dog	Myanmar	1999
409	KX148249	02006CBG	dog	Cambodia	1998
410	KX148250	99016CBG	dog	Cambodia	1999
411	KX148251	99011CBG	dog	Cambodia	1998
412	KX148253	99012CBG	dog	Cambodia	1998
413	KX148254	01016VNM	dog	Viet Nam	2001
414	KX148259	94272PHI	dog	Philippines	1994
415	KX148260	04030PHI	dog	Philippines	2004
416	KX148261	94281PHI	dog	Philippines	1994

417	KX148262	94278PHI	dog	Philippines	1994
418	KX148263	94275PHI	dog	Philippines	1994
419	KX148265	98011CHI	dog	China	1998
420	KX148266	03003INDO	dog	Indonesia	2003
421	KX148267	02050CHI	Homo sapiens	China	1992
422	KX148268	90001FRA	dog	French Guiana	1990
423	KX148269	97004ARG	Tadarida brasiliensis	Argentina	1997
424	KX708499	2041MxYucgod15	dog	Mexico: Yucatan	2015
425	KX708502	5559MxChisdog12	dog	Mexico: Chiapas	2012
426	KX708503	2835MxChisdog14	dog	Mexico: Chiapas	2014
427	KX954123	RUS_FJL_2016	Vulpes lagopus	Russia	2016
428	KY765901	TajikFTA	dog	Tajikistan	2012
429	KY775603	IGU-R201-Nilgai	nilgai	India	2014
430	KY775604	IGU-R202-Mongoose	mongoose	India	2014
431	KY780299	CTMZZ11	Homo sapiens	China	2017
432	LM645015	Gra07.06-1-F	Polar fox	Greenland	2006
433	LM645018	23.06-2-F	Polar fox	Greenland	2006
434	LM645019	03.10-2-F	Polar fox	Greenland	2010
435	LM645020	02.07-3-F	Polar fox	Greenland	2007
436	LM645022	24.06-3-D	Dog	Greenland	2006
437	LM645023	08.06-3-F	Polar fox	Greenland	2006
438	LM645024	10.06-3-F	Polar fox	Greenland	2006
439	LM645027	25.06-3-F	Polar fox	Greenland	2006
440	LM645029	09.07-4-F	Polar fox	Greenland	2007
441	LM645033	02.10-5-F	Polar fox	Greenland	2010

442	LM645034	07.09-6-F	Polar fox	Greenland	2009
443	LM645035	07.10-6-F	Polar fox	Greenland	2010
444	LM645037	10.08-6-F	Polar fox	Greenland	2008
445	LM645039	01.09-7-S	Sheep	Greenland	2009
446	LM645040	02.09-7-S	Sheep	Greenland	2009
447	LM645042	03.09-7-F	Polar fox	Greenland	2009
448	LM645045	05.07-7-F	Polar fox	Greenland	2007
449	LM645046	06.06-7-C	Cat	Greenland	2006
450	LM645047	13.08-7-F	Polar fox	Greenland	2008
451	LM645048	18.05-7-F	Polar fox	Greenland	2005
452	LM645049	3a.07-7-F	Polar fox	Greenland	2007
453	LM645050	3b.07-7-F	Polar fox	Greenland	2007
454	LM645051	01.06-7-F	Polar fox	Greenland	2007
455	LM645052	02.06-7-F	Polar fox	Greenland	2006
456	LM645053	04.07-7-F	Polar fox	Greenland	2007
457	LM645054	15.07-7-S	Sheep	Greenland	2007
458	LM645055	21.06-7-F	Polar fox	Greenland	2006
459	LM645056	02.08-7-F	Polar fox	Greenland	2008
460	LN879480	5989/lib205/12	Canis lupus familiaris	Azerbaijan	2002
461	LN879481	148/lib01041	Vulpes vulpes	Germany	1998

B. Characteristic of ABLV genomes analyzed in present study.

No	Accession	Name	Host	Country	Year
1	AF418014	Australian bat lyssavirus	Homo sapiens		
2	KJ685548	Australian bat lyssavirus H2	Equus ferus caballus (horse)	Australia	2013
3	KT868953	Australian bat lyssavirus 1510	Pteropus alecto	Australia	2014
4	KT868954	Australian bat lyssavirus 2549	Pteropus alecto	Australia	2014
5	KT868955	Australian bat lyssavirus 2566	Pteropus alecto	Australia	2014
6	KT868956	Australian bat lyssavirus 2430	Pteropus scapulatus	Australia	2014

C. Characteristic of DUVV genomes analyzed in present study.

No	Accession	Name	Host	Country	Year
1	EU293119	Duvenhage virus 86132SA	Homo sapiens	South Africa	1971
2	EU293120	Duvenhage virus 94286SA	Miniopterus	South Africa	1981
3	EU623444	Duvenhage virus DUVVSA06	Homo sapiens	South Africa	
4	JN986749	Duvenhage virus NL07	Homo sapiens	Netherlands	2007

D. Characteristic of EBLV genomes analyzed in present study.

No	Accession	Name	Host	Country	Year
1	LT839608	European bat lyssavirus 1—13424			
2	LT839609	European bat lyssavirus 1—20174			
3	LT839610	European bat lyssavirus 1—976			
4	LT839611	European bat lyssavirus 1—5782			
5	LT839612	European bat lyssavirus 1—5006			
6	LT839613	European bat lyssavirus 1—13027			
7	LT839614	European bat lyssavirus 1—5776			
8	LT839615	European bat lyssavirus 1—13454			
9	EF157976	European bat lyssavirus 1—RV9	Eptesicus serotinus	Germany	1968
10	EU293109	European bat lyssavirus 1—03002FRA	Eptesicus serotinus	France	2003

11	EU293112	European bat lyssavirus 1—8918FRA	Eptesicus serotinus	France	1989
12	EU626551	European bat lyssavirus 1—08120FRA	Eptesicus serotinus	France	2008
13	EU626552	European bat lyssavirus 1—07240FRA	cat	France	2007
14	KF155003	European bat lyssavirus 1—RV20	Eptesicus serotinus	Denmark	1986
15	KP241939	European bat lyssavirus 1—RV2416	Eptesicus isabellinus	Spain	2007

E. Characteristic of LBV genomes analyzed in present study.

No	Accession	Name	Host	Country	Year
1	EU259198	Lagos bat virus KE131	Eidolon helvum	Kenya	
2	EU293108	Lagos bat virus 0406SEN	Eidolon helvum	Senegal	1985
3	EU293110	Lagos bat virus 8619NGA	Eidolon helvum	Nigeria	1956
4	GU170202	Lagos bat virus KE576	Rousettus aegyptiacus	Kenya	
5	JX901139	Lagos bat virus KE476	Rousettus aegyptiacus	Kenya	2010
6	LN849915	Lagos bat virus 31225	Eidolon helvum	Ghana:Kumasi	

F. Characteristic of MOKV genomes analyzed in present study.

No	Accession	Name	Host	Country	Year
1	NC_006429	Mokola virus			
2	EU293117	Mokola virus 86100CAM	shrew	Cameroon	1974
3	EU293118	Mokola virus 86101RCA	rodent	Central African Republic	1981
4	KF155006	Mokola virus RV1035	feline	Zimbabwe	1982
5	KF155007	Mokola virus RV1017	feline	Zimbabwe	1993
6	KF155008	Mokola virus RV1021	feline	South Africa	1996
